# Cognitive-Enhancing and Neuroprotective Effects of a Combination Extract of *Atractylodes macrocephala* and *Rumex acetosa* Against Scopolamine-Induced Memory Impairment

**DOI:** 10.3390/ijms27219410

**Published:** 2026-10-22

**Authors:** Dool-Ri Oh, Hwan Lee, Yonguk Kim, Yujin Kim, Donghyuck Bae

**Affiliations:** Natural Resources Research Laboratory, Green Bio Division, Jeonnam Bio Foundation, Jangheung-gun 59338, Republic of Korea; pp0806@nate.com (D.-R.O.); hwanlee@jbf.kr (H.L.); kyu9801@jbf.kr (Y.K.); ngel2327@jbf.kr (Y.K.)

**Keywords:** *Atractylodes macrocephala*, *Rumex acetosa*, scopolamine, neuroprotection, cognition

## Abstract

This study investigated the cognitive-enhancing and neuroprotective effects of a mixed extract of *Atractylodes macrocephala* Koidzumi (AM) and *Rumex acetosa* L. (RA) in models of scopolamine (SCO)-induced toxicity and memory impairment. In vitro, the 2:1 ratio maximized neuroprotection and p-CREB/CREB expression in SCO-treated SH-SY5Y cells. In vivo, rats received the AR mixture orally for 29 days, with SCO challenges introduced from Day 22. In the Morris water maze, the AR mixture reduced escape latency during the 3-day spatial training period and increased target quadrant exploration during the probe trial. In the passive avoidance test, it significantly prolonged step-through latency, confirming enhanced memory retention. Hippocampal biochemical assessments revealed that the AR mixture attenuated acetylcholinesterase activity, decreased malondialdehyde accumulation, and replenished acetylcholine levels. Furthermore, the formulation suppressed the pro-inflammatory cytokines IL-1β, TNF-α, and IL-6. AR mixture treatment attenuated the SCO-associated reductions in BDNF, p-TrkB, p-CREB, and PSD-95 expression and in ERK1/2 and Akt phosphorylation in the hippocampus. These findings suggest that the AR mixture improves cognitive and biochemical outcomes in SCO-treated rats, accompanied by changes in cholinergic function, lipid peroxidation, inflammation, and BDNF/TrkB-related signaling.

## 1. Introduction

Alzheimer’s disease (AD) is a progressive neurodegenerative disorder in which deterioration of memory and other cognitive functions represents a major clinical feature [1]. Among the mechanisms implicated in cognitive dysfunction, disruption of cholinergic neurotransmission is particularly relevant because acetylcholine (ACh) plays an important role in learning, spatial processing, and memory formation [2]. Excessive acetylcholinesterase (AChE) activity can further compromise cholinergic signaling by accelerating ACh degradation in the hippocampus [3]. In addition to cholinergic deficits, oxidative damage and neuroinflammatory responses contribute to AD-related neuronal dysfunction. These processes include lipid peroxidation, commonly assessed by malondialdehyde (MDA), and increased production of inflammatory mediators such as interleukin-1 beta (IL-1β), interleukin-6 (IL-6), and tumor necrosis factor-alpha (TNF-α) [4]. Thus, compounds capable of acting simultaneously on cholinergic dysfunction, oxidative stress, and neuroinflammation are of interest as potential approaches for mitigating cognitive impairment [5].

Pharmacological blockade of muscarinic acetylcholine receptors by scopolamine (SCO) induces acute and reversible cholinergic dysfunction accompanied by learning and memory deficits and is therefore frequently used to establish experimental models of cognitive impairment [6]. Although useful for investigating cholinergic dysfunction and associated cognitive deficits, the SCO model does not reproduce the progressive and multifactorial neuropathology of Alzheimer’s disease (AD). The cognitive alterations induced by SCO are accompanied by disturbances in cholinergic neurotransmission and synaptic plasticity, together with increased oxidative and inflammatory responses [7,8]. Neurotrophic signaling is also closely associated with the maintenance of neuronal function and memory processes. In particular, brain-derived neurotrophic factor (BDNF) activates its high-affinity receptor, tropomyosin receptor kinase B (TrkB), thereby engaging intracellular signaling cascades involving extracellular signal-regulated kinase 1/2 (ERK1/2) and protein kinase B (Akt) [9,10]. These downstream pathways influence cAMP-response element-binding protein (CREB) and synaptic proteins such as postsynaptic density protein-95 (PSD-95), which participate in neuronal survival, synaptic plasticity, and memory consolidation [11].

Natural products containing diverse bioactive constituents provide an opportunity to modulate multiple biological processes associated with cognitive dysfunction. *Atractylodes macrocephala* Koidzumi (AM) has traditionally been used in Eastern medicine to treat gastrointestinal disorders and reportedly has anti-inflammatory, antioxidant, and neuroprotective properties associated with sesquiterpenoids, particularly atractylenolides I and III, and polysaccharides [12]. Similarly, *Rumex acetosa* L. (RA), a perennial herb in the Polygonaceae family, has traditionally been used to clear heat, promote detoxification, and cool the blood to control bleeding [13]. Pharmacological studies have reported antioxidant, anti-inflammatory, and antiproliferative activities of RA [14,15], as well as vascular and platelet-related effects [16,17]. Its aerial parts contain various secondary metabolites, including the flavonoid polyphenols hyperin and vitexin, which have been linked to neuroprotective and cognition-related activities in experimental studies [18,19]. In our previous work, RA improved spatial memory and influenced NR2 subunits, GluA1 receptors, and BDNF/CREB signaling under normal conditions [20]. We subsequently found that RA exerted anti-inflammatory and antiapoptotic effects in SCO-induced cognitive impairment [21]. However, the combined effects of AM and RA (AR mixture) and the associated mechanisms in SCO-induced cognitive impairment remain unclear.

Therefore, this study compared different AM:RA ratios and evaluated the effects of the selected combination extract (AR mixture) on SCO-induced cytotoxicity and memory impairment. In vitro, we compared the protective effects of the individual extracts and their 1:1, 1:2, and 2:1 mixtures (AM:RA) and assessed p-CREB/CREB expression in SCO-treated SH-SY5Y neuroblastoma cells. Based on the in vitro findings, Sprague–Dawley (SD) rats received the 2:1 AR mixture orally for 29 days, and spatial and associative memory were assessed using the Morris water maze (MWM) and passive avoidance (PA) tests after SCO administration. We also measured hippocampal AChE activity, ACh and MDA levels, proinflammatory cytokines, synaptic proteins, and components of BDNF/TrkB-related signaling.

## 2. Results

### 2.1. Simultaneous HPLC Analysis of Four Marker Compounds in the AR Mixture

HPLC-PDA was used to characterize the AR mixture based on four selected marker compounds. The RA-derived compounds 1-caffeoyl-β-D-glucose, quercetin 3-O-robinobioside, and hyperoside (hyperin) were selected as RA markers, whereas the sesquiterpenoid atractylenolide III was selected as the AM marker. All three RA markers and atractylenolide III were detected in the AR mixture (Figure 1A), with retention times corresponding to those of their respective reference standards (Figure 1B–E). These findings confirmed the detectable presence of the four selected marker compounds in the final AR mixture.

### 2.2. Protective Effects of AM, RA, and Their Mixtures Against SCO-Induced Cytotoxicity in SH-SY5Y Cells

To identify an effective combination ratio, the protective effects of AM, RA, and their mixtures (AM:RA ratio, AR mixture) were evaluated in SH-SY5Y cells. First, the cells were exposed to SCO at 0.1–5 mM for 24 h to establish a cytotoxicity model. SCO reduced cell viability in a concentration-dependent manner, with 5 mM SCO decreasing viability to 57.49 ± 0.14% of the control value; this concentration was therefore used in the subsequent experiments (Figure 2A). To assess their intrinsic cytotoxicity, the cells were treated with RA, AM, or each AR mixture at 50 μg/mL for 24 h in the absence of SCO. The mixtures contained AM and RA at weight ratios of 1:1, 1:2, or 2:1. Neither the individual extracts nor their mixtures significantly affected cell viability relative to the control (Figure 2B). Subsequently, the protective effects against SCO-induced cytotoxicity were evaluated by pretreating the cells with RA, AM, or each AR mixture at 30 or 50 μg/mL (Figure 2C). Exposure to 5 mM SCO reduced cell viability to 57.76 ± 1.85% of the control value, whereas pretreatment with the extracts or mixtures significantly attenuated this reduction. Among the tested mixtures, the 2:1 AR mixture produced the greatest protective effect, resulting in cell viability of 109.38 ± 0.62% at 50 μg/mL. At the same concentration, the protective effect of the 2:1 mixture was greater than those of AM or RA alone and the 1:1 and 1:2 mixtures.

To further characterize the molecular response associated with the protective effects, total and phosphorylated CREB levels were assessed at 30 μg/mL, a concentration that retained protective activity while allowing clearer differentiation among the tested formulations (Figure 2D,E). SCO exposure significantly decreased the phosphorylated CREB (p-CREB)/total CREB ratio relative to the control group. Pretreatment with AM partially attenuated this decrease, whereas the 2:1 AR mixture produced the greatest increase in the p-CREB/CREB ratio and had a significantly greater effect than the individual extracts. Together, these findings identified the 2:1 AR mixture as the best-performing ratio among the formulations tested under the present experimental conditions, based on both cell viability and CREB phosphorylation, and this ratio was therefore selected for subsequent in vivo experiments.

### 2.3. Attenuating Effects of the AR Mixture on SCO-Induced Spatial Learning and Memory Impairments in the MWM Test

Spatial learning and memory were evaluated using the MWM to determine whether the protective effects observed in vitro were accompanied by behavioral effects in vivo (Figure 3). During spatial acquisition training, escape latency was analyzed separately for the first and last trials on each of 3 consecutive days. In the first trial of each day, the SCO control group had a significantly longer escape latency than the normal group, indicating impaired spatial learning. Oral administration of the AR mixture attenuated this impairment, with the 50 and 100 mg/kg AR mixture groups showing a significant decrease in escape latency over the 3 training days (Figure 3A). In the last trial of each day, the 50 and 100 mg/kg AR mixture groups also showed a reduced escape latency (Figure 3B). On day 29, the platform was removed for a probe trial assessing spatial memory retention. Representative swimming trajectories showed that the SCO control rats searched broadly across the pool, whereas the AR mixture-treated rats, particularly those receiving 50 and 100 mg/kg, concentrated their search near the former platform location (Figure 3C).

The quantitative probe-trial findings were consistent with these representative trajectories. Compared with the normal group, the SCO control group traveled a shorter distance and spent less time in the target quadrant. Treatment with the AR mixture significantly increased both target-quadrant measures relative to SCO alone. The AR mixture group showed an increased distance traveled in the target quadrant (Figure 3D) and significantly increased target-quadrant dwell time (Figure 3E); the corresponding values were numerically higher than those in the donepezil group. These findings indicate that the AR mixture attenuated SCO-induced deficits in spatial learning and memory.

### 2.4. Effects of the AR Mixture on SCO-Induced Passive Avoidance Impairment

The PA test was performed to evaluate the effect of the AR mixture on SCO-induced impairment of aversive memory retention (Figure 4). During the retention test, the SCO control group had a significantly shorter step-through latency than the normal group (102.11 ± 12.41 vs. 287.88 ± 4.95 s). This reduction indicated impaired passive avoidance memory. Oral administration of the AR mixture attenuated the SCO-induced reduction in step-through latency. Step-through latency was significantly longer in the 50- and 100-mg/kg AR mixture groups than in the SCO control group (279.52 ± 3.79 and 259.55 ± 9.50 s, respectively). These results indicate that the AR mixture attenuated SCO-induced impairment of passive avoidance memory.

### 2.5. Effects of the AR Mixture on SCO-Induced Hippocampal MDA Accumulation

Hippocampal MDA levels were measured using a TBARS assay to evaluate the effect of the AR mixture on SCO-induced lipid peroxidation (Figure 5). SCO significantly increased hippocampal MDA levels relative to the normal group (238.60 ± 4.77 vs. 190.31 ± 2.35 µM/mg protein), indicating increased lipid peroxidation. Oral supplementation of the AR mixture attenuated this increase. MDA levels were significantly lower in the 50 and 100 mg/kg AR mixture groups than in the SCO control group (185.31 ± 3.17 and 189.58 ± 2.34 µM/mg protein, respectively; *p* < 0.05).

### 2.6. Regulatory Effects of the AR Mixture on SCO-Induced Hippocampal Cholinergic Dysfunction

Hippocampal AChE activity and ACh concentrations were measured to evaluate the effect of the AR mixture on cholinergic function (Figure 6). SCO significantly increased AChE activity (*p* < 0.05; Figure 6A) and decreased ACh concentration (*p* < 0.01; Figure 6B) relative to the normal group. Compared with SCO alone, the AR mixture significantly reduced AChE activity at 50 and 100 mg/kg (*p* < 0.05 and *p* < 0.01, respectively; Figure 6A) and increased ACh concentrations (*p* < 0.01; Figure 6B). These findings suggest that modulation of hippocampal cholinergic function may contribute to the behavioral effects of the AR mixture.

### 2.7. Effects of the AR Mixture on SCO-Induced Hippocampal Neuroinflammation

Hippocampal concentrations of the proinflammatory cytokines IL-1β (Figure 7A), IL-6 (Figure 7B), and TNF-α (Figure 7C) were measured by ELISA to evaluate the effects of the AR mixture on neuroinflammation. SCO administration significantly increased all three cytokines relative to the normal group, and the AR mixture attenuated these increases. At 50 and 100 mg/kg, the AR mixture significantly reduced hippocampal concentrations of all three cytokines relative to SCO alone. These findings indicate that AR mixture treatment was associated with attenuation of the SCO-induced hippocampal inflammatory response.

### 2.8. Effects of the AR Mixture on Hippocampal BDNF/TrkB/CREB Signaling and Synaptic Plasticity-Related Proteins

To investigate potential molecular mechanisms underlying the effects of the AR mixture, proteins involved in neurotrophic signaling, synaptic plasticity, and cell-survival pathways were evaluated (Figure 8). Compared with the normal group, the SCO control group had significantly lower hippocampal BDNF levels (*p* < 0.05), TrkB phosphorylation (*p* < 0.001), and CREB phosphorylation (*p* < 0.01). The AR mixture significantly increased the BDNF/β-actin ratio (*p* < 0.05 or *p* < 0.01), p-TrkB/TrkB ratio (*p* < 0.001), and p-CREB/CREB ratio (*p* < 0.01 or *p* < 0.001) relative to SCO alone. SCO also significantly reduced hippocampal PSD-95 expression (*p* < 0.01), ERK1/2 phosphorylation (*p* < 0.05), and Akt phosphorylation (p < 0.001). The AR mixture significantly increased PSD-95 expression (*p* < 0.05), the p-ERK1/2/ERK1/2 ratio (*p* < 0.05), and the p-Akt/Akt ratio (*p* < 0.001) relative to SCO alone.

## 3. Discussion

The present study showed that a 2:1 mixture of *Atractylodes macrocephala* Koidzumi and *Rumex acetosa* L. (AR mixture) attenuated SCO-induced cognitive impairment and associated biochemical alterations. We initially used SH-SY5Y neuroblastoma cells to compare several AM:RA ratios and found that the 2:1 mixture produced the greatest protective effect among the tested formulations. On the basis of these in vitro findings, the 2:1 mixture was selected for the in vivo experiments, in which its behavioral and hippocampal biochemical effects were evaluated. Oral administration of the AR mixture reduced hippocampal inflammatory cytokine and MDA levels, altered BDNF/TrkB/CREB signaling, and increased PSD-95 expression and ERK1/2 and Akt phosphorylation in SCO-treated rats.

SCO induces acute and reversible cognitive deficits primarily associated with cholinergic dysfunction and impaired synaptic plasticity [6]. Accordingly, the present findings should be interpreted as evidence that the AR mixture attenuates SCO-induced cognitive impairment rather than as evidence of a disease-modifying effect on AD pathology. In the MWM, oral administration of the AR mixture reduced escape latency during the 3-day spatial training period and increased target-quadrant occupancy, indicating attenuation of SCO-induced spatial learning and memory deficits. Consistent with the MWM findings, the AR mixture prolonged step-through latency in the PA test. Together, these behavioral findings indicate that the AR mixture attenuated SCO-induced impairment of spatial and passive avoidance memory.

Central cholinergic dysfunction, including alterations in acetylcholine release and receptor expression, is closely associated with cognitive impairment [22]. AR mixture administration reduced hippocampal AChE activity, increased hippocampal ACh concentrations, and reduced hippocampal MDA levels, a marker of lipid peroxidation associated with SCO-induced oxidative stress, relative to SCO treatment alone. Donepezil, a clinically used acetylcholinesterase inhibitor, was included as a positive control for SCO-induced cognitive impairment. However, as donepezil primarily provides symptomatic cholinergic enhancement, its use as a comparator does not indicate therapeutic equivalence or a shared mechanism of action with the AR mixture. Accordingly, the effects of the AR mixture were interpreted primarily in comparison with the SCO control group.

The concurrent reductions in hippocampal inflammatory cytokine and MDA levels indicate that AR mixture treatment was associated with multiple biochemical changes in SCO-treated rats. Although the reduction in MDA levels suggests attenuation of lipid peroxidation, MDA alone is insufficient to elucidate the antioxidant mechanisms of the AR mixture. Further studies incorporating additional oxidative stress and antioxidant defense markers are therefore needed to clarify its effects on oxidative stress pathways. In addition, pro-inflammatory cytokines can suppress BDNF/TrkB signaling and impair synaptic plasticity [23], suggesting a potential relationship between the reduced inflammatory response and neurotrophic signaling observed in the present study. AR mixture treatment increased BDNF expression, the p-TrkB/TrkB ratio, ERK1/2 and Akt phosphorylation, CREB phosphorylation, and PSD-95 expression relative to SCO treatment alone. Because BDNF/TrkB signaling and PSD-95 contribute to synaptic plasticity and receptor anchoring at the postsynaptic density [24], these changes may be associated with improved synaptic function following AR mixture treatment. Recent studies have highlighted the involvement of oxidative stress, mitochondrial dysfunction, and neuroinflammatory processes in neurodegenerative conditions. For example, Yong et al. reported that Thonningianin A attenuated neuronal ferroptosis and lipid peroxidation through GPX4-related signaling in experimental AD models [25]. Similarly, Guo et al. demonstrated that Ciliatoside A reduced neuroinflammation in association with enhanced mitophagy and reduced NLRP3 inflammasome activation, while Zhou et al. discussed the relationship between impaired mitophagy and inflammatory signaling in neurodegenerative conditions [26,27]. However, the involvement of these specific mechanisms in the effects of the AR mixture remains to be investigated.

The AR mixture contains diverse phytochemicals derived from both plant sources. AM contains sesquiterpenoids such as atractylenolides I, II, and III, which have demonstrated anti-inflammatory, antioxidant, and neuroprotective activities [28]. RA contains anthraquinones and flavonoids, including hyperin, vitexin, and proanthocyanidins, with reported antioxidant, anti-inflammatory, and neuroprotective activities [13,29,30,31]. However, the present study did not determine the concentrations or individual biological activities of these constituents in the administered AR mixture, and therefore, the observed behavioral and biochemical effects cannot be attributed to any specific constituent. Although the chemical profile of the AR mixture was characterized by HPLC-PDA, quantitative standardization data, including the contents of the four marker compounds and the extract-to-dextrin ratio in the final preparation, were not reported in the present study. Further quantitative characterization and evaluation of isolated constituents and defined combinations will be needed to identify the compounds contributing to the observed effects and their potential pharmacological interactions. In addition, because the in vivo study did not include groups treated with AM or RA alone, the effects of the individual extracts and their potential additive or synergistic interactions remain to be determined. Pathway-specific inhibition, activation, or rescue experiments will also be needed to further clarify the involvement of BDNF/TrkB/CREB, ERK/Akt, and related synaptic signaling in the effects of the AR mixture.

## 4. Materials and Methods

### 4.1. Chemicals and Reagents

WST-1, SCO, β-actin, atractylenolide III, and hyperoside were purchased from Sigma Chemical Co. (St. Louis, MO, USA). 1-caffeoyl-β-D-glucose was purchased from Biobiopha Co., Ltd. (Kunming, China). Quercetin 3-O-robinobioside was purchased from ChemFaces.Cn. (Wuhan, China). Amplex™ Red Acetylcholine/Acetylcholinesterase assay kit, minimum essential medium (MEM), and fetal bovine serum (FBS) were purchased from Invitrogen (Grand Island, NY, USA). The Syn-PER Synaptic Protein Extraction Reagent and BCA Protein Assay Reagent were purchased from Thermo Fisher Scientific (Waltham, WA, USA). Anti-PSD-95, anti-t-TrkB, anti-phospho-TrkB, anti-t-CREB, anti-phospho-CREB, anti-t-ERK, anti-phospho-ERK, anti-t-Akt, anti-phospho-Akt, horseradish peroxidase (HRP)-conjugated anti-rabbit and anti-mouse immunoglobulin (Ig)G antibody were purchased from Cell Signaling Technology (Boston, MA, USA). Anti-BDNF antibody was purchased from Santa Cruz Biotechnology (Dallas, TX, USA). The rat IL-1β, IL-6, and TNF-α ELISA kit was purchased from R&D Systems (Minneapolis, MN, USA). The MDA assay kit was purchased from Cell BioLabs Inc. (San Diego, CA, USA). HPLC-grade acetonitrile and water were purchased from J.T.Baker (Phillipsburg, NJ, USA).

### 4.2. Preparation of Extract

AM root was purchased from Kwangdeok (Geumsan-gun, Republic of Korea). RA leaves were prepared and authenticated as described previously [20]. The voucher specimens of AM (No. JINR-A0020) and RA (No. JINR-RA0010) have been deposited in the Natural Resources Research Laboratory, Green Bio Division (Jangheung-gun, Republic of Korea). AM and RA were separately extracted with water at 90–100 °C for 3–4 h, filtered, and concentrated under reduced pressure. The extracts were combined at dry-solid weight ratios of 1:1, 1:2, and 2:1 (AM:RA); the 2:1 ratio (AR mixture) was formulated with dextrin as an excipient into a standardized powder and stored at 4 °C until use.

### 4.3. Isolation of Bioactive Marker Compounds from RA

RA was extracted with distilled water, and the aqueous extract was successively partitioned with *n*-hexane, chloroform, ethyl acetate, and *n*-butanol. Based on preliminary HPLC results, the major bioactive constituents were predominantly distributed in the chloroform fraction (CF). Therefore, CF was selected for further targeted purification. CF was subjected to medium-pressure liquid chromatography (MPLC) on a C18 reversed-phase column using a gradient elution of 10–50% methanol, yielding four subfractions (CF-1–CF-4). Fractions CF-2 and CF-3, which contained the major marker peaks, were further purified via repeated preparative HPLC. Preparative isolation of CF-2 afforded compound **1** (5.0 mg). while subsequent purification of CF-3 yielded compound **2** (23.6 mg) and compound **3** (4.3 mg). The chemical structures of the isolated compounds were definitively elucidated by analyzing one- and two-dimensional NMR spectroscopy (COSY, HSQC, and HMBC) and comparison with published spectroscopic data.

Compound **1** (1-caffeoyl-β-D-glucose): White amorphous powder; ^1^H NMR (500 MHz, CD_3_OD) δ 7.67 (1H, d, *J* = 16.0 Hz, H-7), 7.06 (1H, d, *J* = 2.1 Hz, H-2), 6.98 (1H, dd, *J* = 8.3, 2.0 Hz, H-6), 6.78 (^1^H, d, *J* = 8.0 Hz, H-5), 6.30 (^1^H, d, *J* = 16.0 Hz, H-8), 5.57 (^1^H, d, *J* = 7.8 Hz, H-1′), 3.86 (^1^H, dd, *J* = 12.2, 2.0 Hz, H-6′a), 3.70 (^1^H, dd, *J* = 12.2, 4.8 Hz, H-6′b), 3.48–3.35 (^4^H, m, H-2′–H-5′). ^13^C NMR (125 MHz, CD_3_OD) δ 167.91 (C-9), 150.13 (C-4), 148.51 (C-7), 147.05 (C-3), 127.71 (C-1), 123.39 (C-6), 116.68 (C-8), 115.38 (C-5), 114.51 (C-2), 95.93 (C-1′), 78.97 (C-5′), 78.19 (C-3′), 74.20 (C-2′), 71.27 (C-4′), 62.50 (C-6′). Comparison of the obtained spectroscopic data with those reported in the literature confirmed the compound to be 1-caffeoyl-β-D-glucose [32] (Supplementary Figures S1–S5).

Compound **2** (Quercetin 3-*O*-robinobioside): Yellow amorphous powder; ^1^H NMR (500 MHz, CD_3_OD) δ 7.86 (1H, d, *J* = 2.0 Hz, H-2′), 7.58 (^1^H, dd, *J* = 8.4, 2.0 Hz, H-6′), 6.85 (^1^H, d, *J* = 8.4 Hz, H-5′), 6.39 (^1^H, d, *J* = 2.0 Hz, H-8), 6.19 (^1^H, d, *J* = 2.0 Hz, H-6), 5.06 (^1^H, d, *J* = 7.8 Hz, H-1″), 4.51 (^1^H, d, *J* = 1.5 Hz, H-1‴), 3.84–3.81 (^1^H, m, H-2″), 3.79 (^1^H, dd, *J* = 3.1, 1.3 Hz, H-4″), 3.73 (^1^H, dd, *J* = 10.0, 5.8 Hz, H-6″a), 3.64 (^1^H, td, *J* = 6.2, 1.2 Hz, H-5″), 3.56 (^2^H, m, H-3″ and H-2‴), 3.53 (^1^H, dd, J = 9.6, 3.3 Hz, H-3‴), 3.50–3.46 (^1^H, m, H-5‴), 3.42–3.36 (^2^H, m, H-6″b and H-4‴), 1.17 (^3^H, d, *J* = 6.2 Hz, H-6‴). 13C NMR (125 MHz, CD_3_OD) δ 178.14 (C-4), 164.79 (C-7), 161.62 (C-5), 157.61 (C-9), 157.12 (C-2), 148.69 (C-4′), 144.42 (C-3′), 134.52 (C-3), 121.66 (C-1′), 121.43 (C-6′), 116.62 (C-5′), 114.78 (C-2′), 104.62 (C-1″), 104.21 (C-10), 100.56 (C-1‴), 98.61 (C-6), 93.48 (C-8), 73.93 (C-3″), 73.74 (C-5″), 72.52 (C-4‴), 71.79 (C-2″), 70.92 (C-3‴), 70.71 (C-2‴), 68.84 (C-4″), 68.37 (C-5‴), 65.95 (C-6″), 16.64 (C-6‴). A comparison of the obtained spectroscopic data with those reported in the literature confirmed that the compound was quercetin 3-O-robinobioside [33] (Supplementary Figures S6–S11).

Compound **3** (Hyperoside): Yellow amorphous powder; ^1^H NMR (500 MHz, CD_3_OD) δ 7.83 (^1^H, d, *J* = 2.0 Hz, H-2′), 7.57 (^1^H, dd, *J* = 8.4, 2.0 Hz, H-6′), 6.85 (^1^H, d, *J* = 8.4 Hz, H-5′), 6.39 (^1^H, d, *J* = 2.0 Hz, H-8), 6.19 (^1^H, d, *J* = 2.0 Hz, H-6), 5.15 (^1^H, d, *J* = 7.8 Hz, H-1″), 3.86–3.35 (^6^H, m, H-2″–H-6″). The ^1^H NMR spectroscopic data were consistent with those reported for hyperoside [34] (Supplementary Figure S12).

### 4.4. Simultaneous High-Performance Liquid Chromatography Analysis of Marker Compounds

HPLC-PDA analysis was performed using a Waters e2695 system with a 2998 PDA detector and a Waters XBridge^®^ C18 column (250 × 4.6 mm, 5 μm) at 35 °C, using a gradient of acetonitrile and water (Supplementary Table S1), a flow rate of 1.0 mL/min, and detection at 240 nm. The AR mixture and marker compounds were prepared in 50% methanol at 30 mg/mL and 100 μg/mL, respectively, and filtered (0.45-μm PVDF) before injection.

### 4.5. SH-SY5Y Cell Cultures and Cell Viability Assay

The human neuroblastoma cell line SH-SY5Y was obtained from the American Type Culture Collection (ATCC; CRL-2266; Manassas, VA, USA). The cells were maintained in MEM supplemented with 10% FBS, 100 U/mL penicillin, and 100 µg/mL streptomycin at 37 °C in a humidified atmosphere containing 5% CO_2_. To assess SCO-induced cytotoxicity, undifferentiated SH-SY5Y cells were seeded in 96-well plates at 1.0 × 104 cells/well, cultured for 24 h, and then exposed to various SCO concentrations for 24 h. The SCO concentration that reduced cell viability by approximately 50% (IC_50_) was selected for the subsequent cytotoxicity experiments. To evaluate the intrinsic cytotoxicity of the extracts and mixtures, cells were incubated with RA, AM, or each AM:RA mixture at 50 μg/mL for 24 h in the absence of SCO. To evaluate their protective effects, cells were pretreated with RA, AM, or the AM:RA mixtures at 30 or 50 μg/mL for 2 h and then exposed to SCO (5 mM) for 24 h. After treatment, WST-1 reagent was added to each well to assess cell viability. After incubation for 3 h under standard culture conditions, absorbance was measured at 450 nm using a microplate reader. To correct for potential background absorbance from the extracts, cell-free blank wells containing the corresponding concentrations of each extract or mixture and WST-1 reagent were measured under the same conditions, and the resulting absorbance values were subtracted from the respective sample measurements.

### 4.6. Animals and Experimental Design

Five-week-old male Sprague–Dawley (SD) rats weighing 110–150 g were purchased from Samtako Bio Korea (Osan, Republic of Korea). The rats were housed at 22 ± 2 °C under a 12-h light/dark cycle, with ad libitum access to food and water. The rats were acclimatized for 7 days before the experiments. The animal experiments were approved by the Institutional Animal Care and Use Committee (IACUC) of the Natural Products Center, Green Bio Division, Jeonnam Bio Foundation, on 9 June 2026 (approval no. JINR-2604-2026). The sample size was determined based on our previous study using a comparable SCO-induced cognitive impairment model [21]. The rats were randomly assigned to five groups (n = 7 per group): (1) normal group, vehicle only; (2) SCO control group, SCO at 1 mg/kg; (3) positive-control group, SCO plus donepezil at 2 mg/kg; (4) low-dose group, SCO plus the AR mixture at 50 mg/kg; and (5) high-dose group, SCO plus the AR mixture at 100 mg/kg. During behavioral testing, animals from different experimental groups were assessed in an interspersed order to minimize potential confounding effects related to measurement order. The normal and SCO control groups received equivalent volumes of vehicle (saline). The AR mixture, donepezil or vehicle was administered once daily by oral gavage for 29 days. To induce cognitive impairment, SCO (1 mg/kg, intraperitoneally) was administered once daily on days 22–29, 30 min before behavioral assessments (Figure 9).

### 4.7. MWM Test

To assess spatial learning and memory capacities, the MWM test was performed in a circular water tank (diameter: 180 cm, height: 75 cm). The water temperature within the tank was regulated at 22 ± 3 °C, and a non-toxic white colorant was introduced to render the water opaque. A circular escape platform (10 cm in diameter) was positioned 2 cm beneath the water surface, remaining concealed from the animals’ view. The experimental arena was conceptually partitioned into four symmetrical quadrants (Northeast, Southeast, Northwest, and Southwest). The maze apparatus was housed in a distraction-free environment characterized by regulated lighting (100–300 lx), minimized background noise (<60 dB), and a fixed 12-h light/dark cycle to reduce potential external stress. Before starting the formal acquisition sessions, each rat underwent a habituation trial on Day 25, swimming freely for 1 min without the platform. Spatial acquisition training was then conducted on three consecutive days (days 26–28), with each rat completing four trials per day and up to 4 min to locate the hidden platform; rats that did not find the platform were guided to it and allowed to remain for 20 s. On Day 29, a probe trial was conducted with the platform removed, and each rat was allowed to explore swim freely for 90 s. Spatial memory retention was evaluated by measuring the time spent and distance traveled within the target quadrant. All behavioral paths and relative parameters were captured and analyzed utilizing the SMART v3.0 video tracking system (Panlab, Barcelona, Spain).

### 4.8. PA Test

The PA test was performed using a two-compartment (light and dark) apparatus separated by an automated sliding door. The grid floor of the dark compartment was connected to an adjustable shock generator. The test was executed over three consecutive phases: habituation, training, and retention testing. On the first day (Day 22), each rat underwent a habituation session to acclimate to the apparatus by being allowed to freely explore both compartments for 5 min with the door open. On the second day (Day 23), the training session was conducted; the rat was placed in the illuminated compartment facing away from the dark compartment, and after a 10 s stabilization period, the sliding door was opened. As soon as the rat entered the dark compartment with all four paws, the door was instantly closed, and an electric foot-shock (0.5 mA, 3 s duration) was delivered through the grid floor. On the third day (Day 24), the retention test was performed without any foot-shock. The rat was placed in the light compartment, the door was opened, and the step-through latency to enter the dark compartment was recorded. The maximum cutoff time for the retention test was set at 300 s. All behavioral data, including step-through latencies and shock delivery, were automatically controlled and recorded using the MED-PC IV software (Version 4; MED Associates Inc., St. Albans, VT, USA).

### 4.9. Preparation of the Hippocampal Tissues

Following the completion of the final behavioral assessment and SCO administration on Day 29, the rats were humanely euthanized by gradual CO_2_ inhalation in a dedicated chamber, in strict accordance with the approved institutional animal care guidelines. The hippocampi were rapidly isolated from the brain tissue within a minimal timeframe, immediately frozen in liquid nitrogen, and stored at −80 °C until analysis. The tissue was homogenized on ice in Syn-PER reagent containing 1× protease inhibitor cocktail at 10 µL of reagent per milligram of tissue. The lysates were centrifuged at 1200× *g* for 10 min at 4 °C, and the resulting supernatants were then centrifuged at 15,000× *g* for 20 min. The resulting pellet was resuspended in Syn-PER reagent and stored at −80 °C until use. For biochemical analyses, tissue samples were labeled with numerical codes, and the investigators performing the analyses were blinded to the treatment allocation.

### 4.10. Biochemical Analysis of Cholinergic Function, Oxidative Stress, and Neuroinflammation

Biochemical alterations within the extracted hippocampal fractions were systematically evaluated to quantify cholinergic functions, lipid peroxidation, and neuroinflammatory responses. Total protein content across all processed samples was determined using a BCA Protein Assay Kit to standardize the respective parameters against mg protein levels.

As an index of oxidative stress and lipid peroxidation, MDA accumulation in the hippocampal tissue was evaluated using a thiobarbituric acid reactive substance (TBARS) assay. Briefly, a 100-µL aliquot of each sample was mixed with an equal volume of sodium dodecyl sulfate lysis solution in a microcentrifuge tube and incubated at room temperature for 5 min. Thiobarbituric acid (TBA) reagent (250 µL) was then added to each mixture. The reaction tubes were heated at 95 °C for 60 min, cooled on ice for 5 min, and centrifuged at 3000 rpm for 15 min before the absorbance of the supernatant was measured at 532 nm. MDA concentrations were calculated from a standard curve and expressed as µM per milligram of protein.

The concentrations of the proinflammatory cytokines IL-1β, IL-6, and TNF-α in the hippocampal lysates were measured using rat Quantikine ELISA kits (R&D Systems) according to the manufacturer’s instructions. Absorbance was measured using a microplate reader, and the concentration of each cytokine was expressed as picograms per milligram of protein.

To evaluate cholinergic homeostasis, acetylcholine (ACh) levels and acetylcholinesterase (AChE) activity in the hippocampal lysates were measured using an acetylcholine/acetylcholinesterase assay kit (Invitrogen) according to the manufacturer’s instructions. AChE hydrolyzes ACh to choline, which is oxidized by choline oxidase to produce hydrogen peroxide (H_2_O_2_); in the presence of horseradish peroxidase (HRP), H_2_O_2_ reacts stoichiometrically with Amplex Red reagent (10-acetyl-3,7-dihydroxyphenoxazine) to generate the fluorescent product resorufin. Hippocampal lysates were diluted in the supplied reaction buffer and mixed with a working solution containing Amplex Red reagent, HRP, and choline oxidase; exogenous ACh was also added for the AChE activity assay. Following a designated incubation period at room temperature protected from light, the OD or fluorescence profile was measured using a microplate reader at an absorbance wavelength of 570 nm. AChE activity and ACh concentrations were calculated from standard curves and expressed as U/mg protein and µM/mg protein, respectively.

### 4.11. Western Blot Analysis

Total protein extraction was performed on both harvested in vitro cells and in vivo hippocampal tissues. For the in vitro samples, undifferentiated SH-SY5Y cells were seeded in 60-mm culture dishes, pretreated with the individual extracts or AM:RA mixtures at 30 µg/mL for 2 h, and then exposed to 5 mM SCO for 12 h before lysis in ice-cold RIPA buffer containing protease and phosphatase inhibitors. For the in vivo samples, the previously prepared hippocampal tissue fractions were utilized. Protein content was determined using the BCA protein assay reagent, with bovine serum albumin (Sigma–Aldrich, St. Louis, MO, USA) used as the standard. Protein lysates were separated on sodium dodecyl sulfate-polyacrylamide gels using a PowerPac^TM^ Basic electrophoresis apparatus (Bio-Rad, Hercules, CA, USA). After transfer, the membranes were blocked for 2 h at 21 ± 2 °C in a blocking solution [1 × Tris-buffered saline containing 0.2% Tween-20 and 5% skimmed milk]. Furthermore, immunoblotting was performed overnight at 4 °C using PSD-95, t-TrkB, phospho-TrkB, t-CREB, phospho-CREB, t-ERK, phospho-ERK, t-Akt, phospho-Akt, BDNF, and β-actin antibodies. Subsequently, the membranes were washed five times with washing solution (1 × TBS containing 0.2% Tween-20) and incubated with diluted HRP-labeled anti-rabbit or anti-mouse IgG for 1 h at room temperature. Immunoreactive bands were visualized using SuperSignal™ West Pico PLUS Chemiluminescent Substrate and a ChemiDoc™ MP Imaging System (Bio-Rad).

### 4.12. Statistical Analysis

Data are presented as the mean ± standard error of the mean (SEM). Statistical analyses were performed using the one-way analysis of variance (ANOVA) with GraphPad Prism version 10 for Windows (GraphPad Software, San Diego, CA, USA). Significant differences between groups were assessed using Tukey’s multiple-comparisons test to account for multiple pairwise comparisons. The normality of residuals and homogeneity of variances were assessed using the Shapiro–Wilk and Brown–Forsythe tests, respectively. Statistical significance was set at *p* < 0.05. The results of the statistical assumption tests, ANOVA statistics, and Tukey-adjusted p-values for selected pairwise comparisons are provided in Supplementary Table S2.

## 5. Conclusions

In conclusion, the 2:1 AR mixture attenuated SCO-induced cognitive impairment and was associated with changes in hippocampal cholinergic function, lipid peroxidation, inflammatory cytokines, and BDNF/TrkB/ERK/Akt/CREB signaling in rats. Further studies should identify and standardize the bioactive constituents, formally evaluate their interactions, and assess the formulation in chronic and disease-relevant models of neurodegeneration.

## Figures and Tables

**Figure 1:**
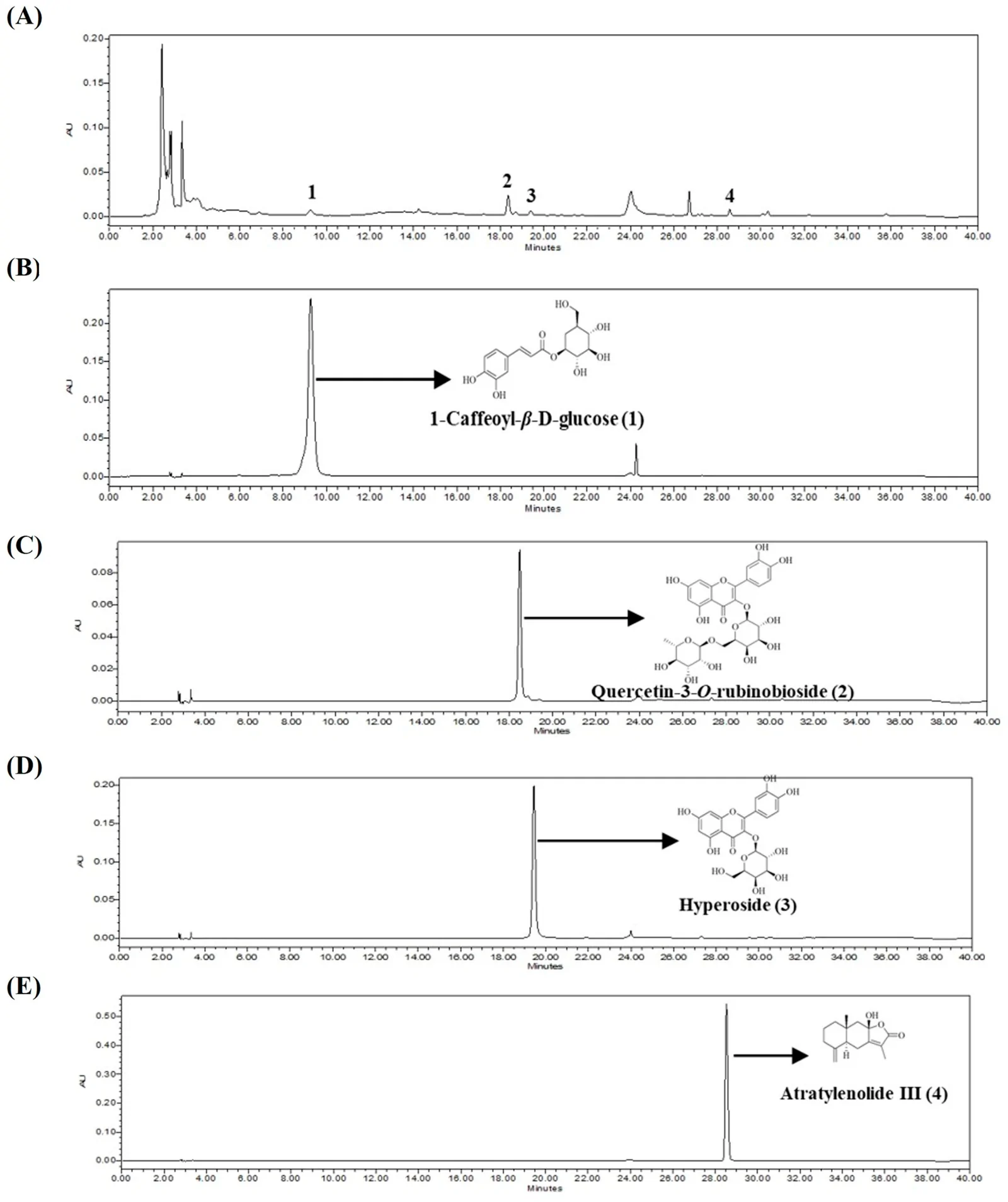
HPLC chromatograms of the AR mixture and its major marker compounds recorded at 240 nm. (**A**) AR mixture extract (AM:RA = 2:1), (**B**) 1-caffeoyl-β-D-glucose (peak 1; *t_R_* = 9.31 min), (**C**) quercetin 3-O-robinobioside (peak 2; *t_R_* = 18.48 min), (**D**) hyperoside (peak 3; *t_R_* = 19.52 min), and (**E**) atractylenolide III (peak 4; *t_R_* = 28.61 min).

**Figure 2:**
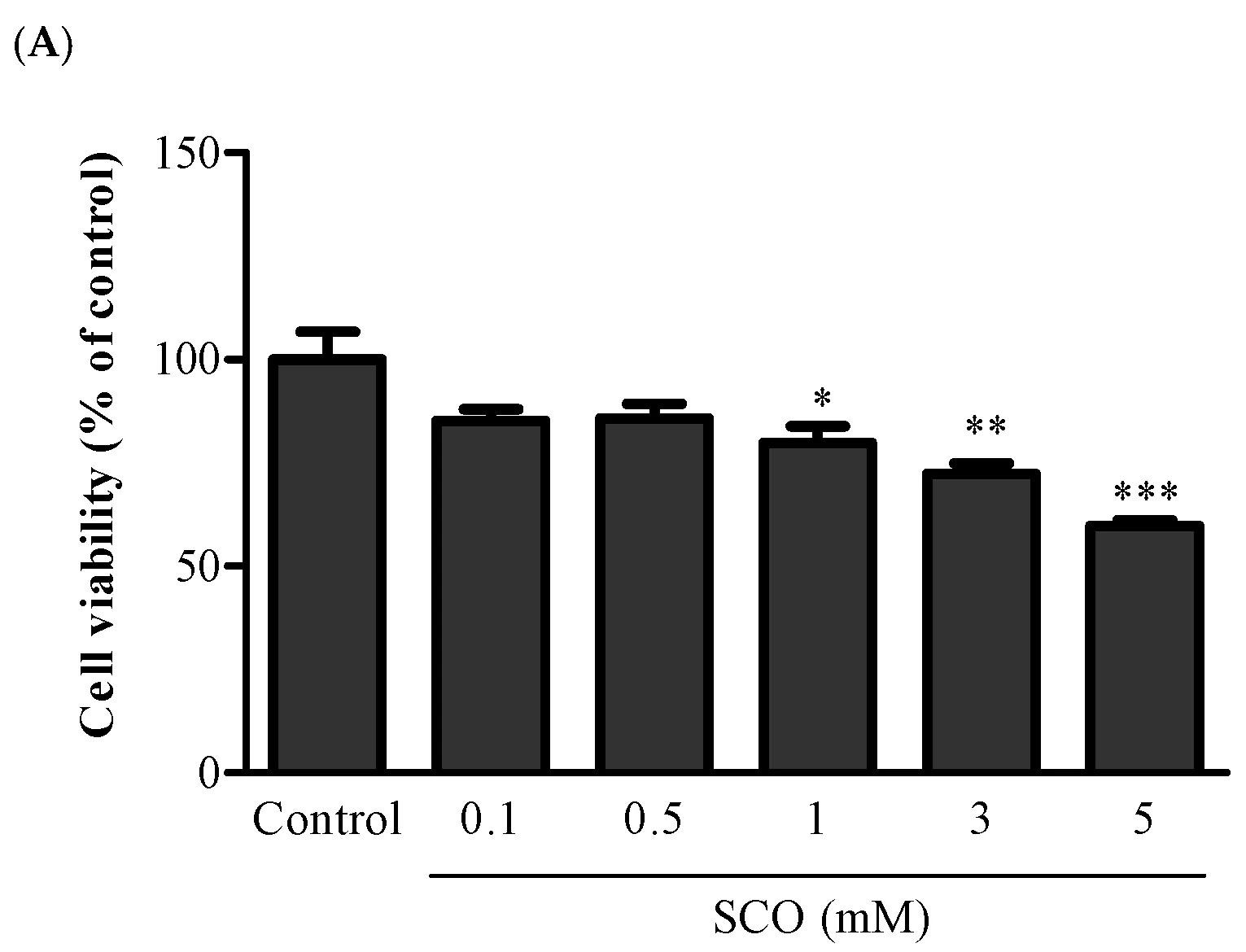

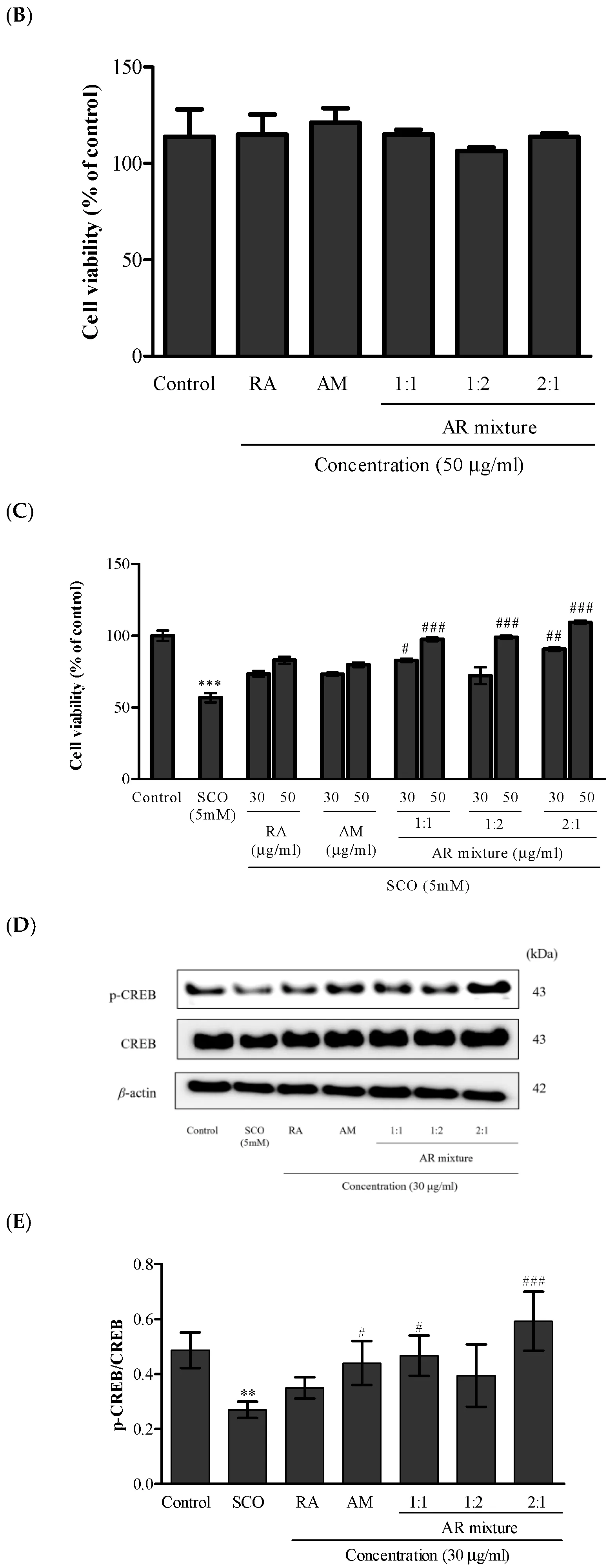
Effects of AM, RA, and their mixtures on SCO-induced cytotoxicity and CREB phosphorylation in SH-SY5Y cells. (**A**) Concentration-dependent effect of SCO on cell viability. SH-SY5Y cells were treated with different concentrations of SCO for 24 h, and cell viability was measured using the WST-1 assay. (**B**) Cytotoxicity screening of individual extracts and different mixing ratios for 24 h. (**C**) Effects of pretreatment with AM, RA, and the AM:RA mixtures (AR mixtures) on SCO-induced reductions in cell viability. Cells were pretreated with AM, RA, or the AR mixtures (1:1, 1:2, or 2:1) at 30 or 50 μg/mL for 2 h and then exposed to 5 mM SCO for 24 h. Cell viability was quantified using the WST-1 assay. (**D**) Representative Western blots of phosphorylated CREB (p-CREB), total CREB (t-CREB), and β-actin. (**E**) Densitometric analysis of the p-CREB/t-CREB ratio. The values are expressed as the mean ± standard error of the mean (*n* = 3). * *p* < 0.05, ** *p* < 0.01 and *** *p* < 0.001 compared with the control group; # *p* < 0.05, ## *p* < 0.01 and ### *p* < 0.001 compared with the SCO group. AM, *Atractylodes macrocephala* Koidzumi; AR mixture, *Atractylodes macrocephala* Koidzumi. and *Rumex acetosa* L.; CREB, cAMP-response element-binding protein; SCO, scopolamine.

**Figure 3:**
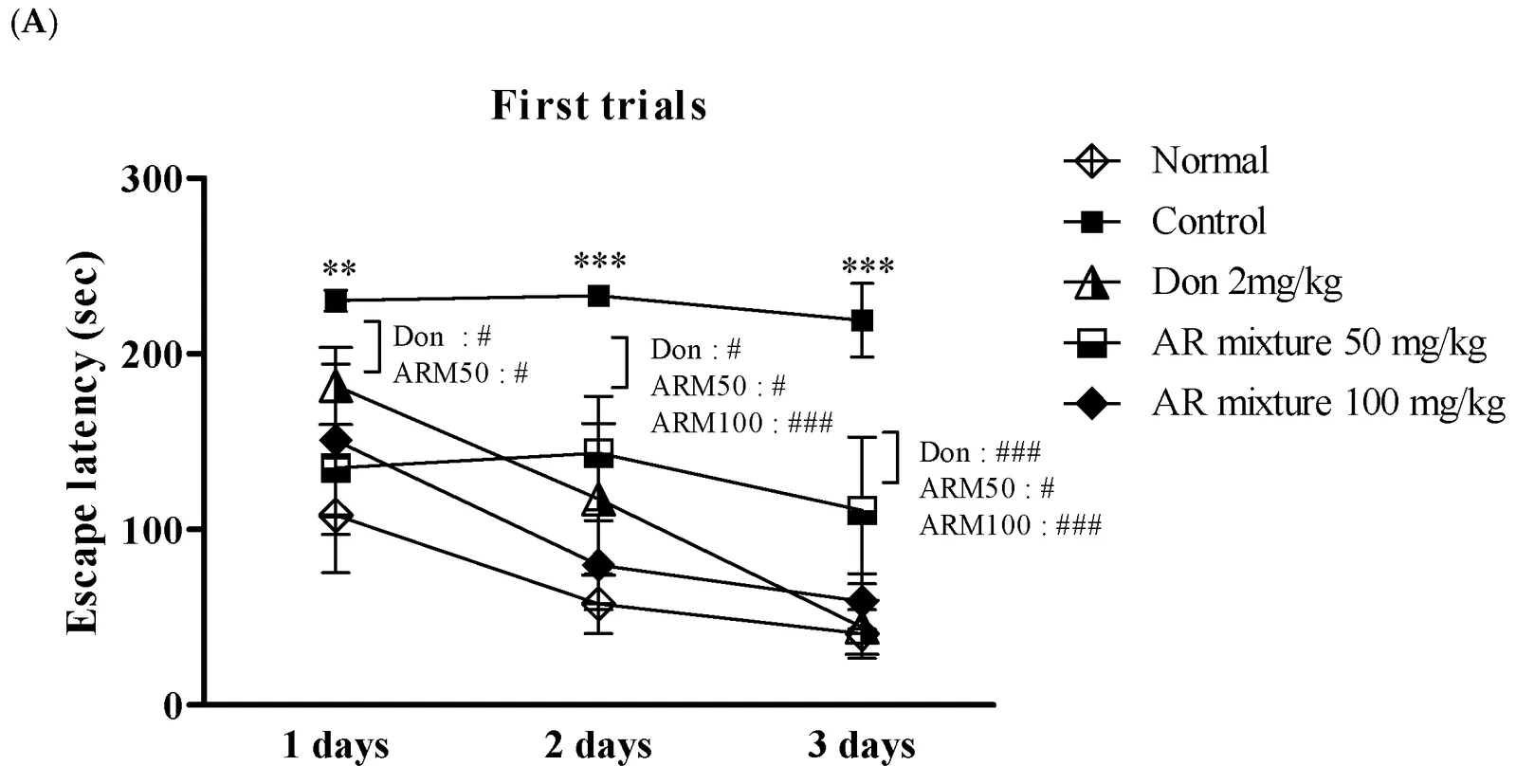

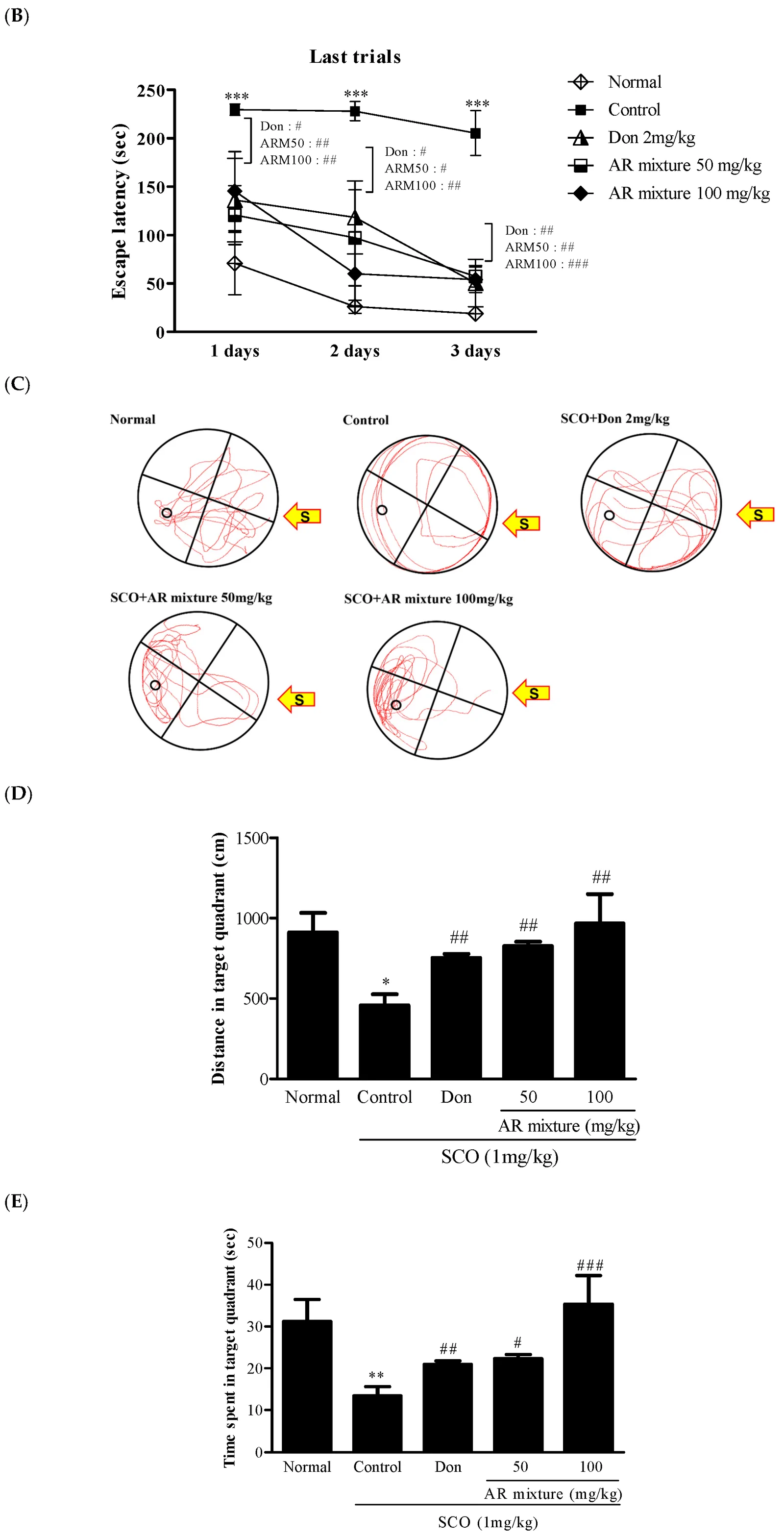
Effects of the AR mixture on SCO-induced spatial learning and memory impairment in the Morris water maze. Escape latency (s) during the first (**A**) and last (**B**) trials of each training day. (**C**) Representative swimming trajectories during the probe trial. The arrow labeled “S” indicates the starting position, and the colored lines represent the swimming trajectories. Distance traveled in the target quadrant (**D**) and time spent in the target quadrant (**E**) during the probe trial. Data are presented as the mean ± SEM (*n* = 7 rats per group). * *p* < 0.05, ** *p* < 0.01 and *** *p* < 0.001 compared with the normal group; # *p* < 0.05, ## *p* < 0.01 and ### *p* < 0.001 compared with the SCO-only control group. AR mixture, *Atractylodes macrocephala* Koidzumi and *Rumex acetosa* L. at a 2:1 ratio; Don, donepezil 2 mg/kg; MWM, Morris water maze; SCO, scopolamine.

**Figure 4:**
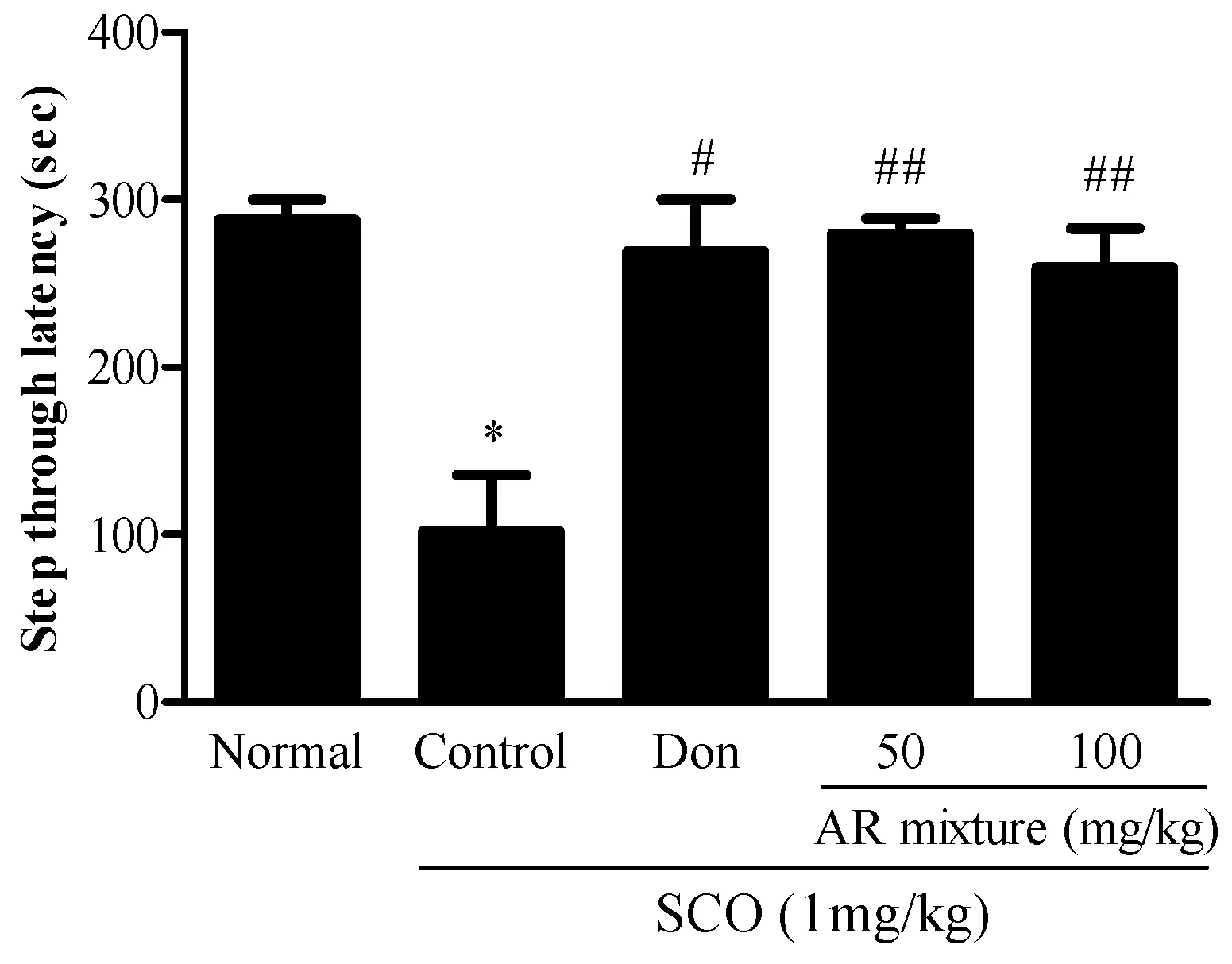
Effects of the AR mixture on SCO-induced passive avoidance impairment. Step-through latency (s) during the retention trial conducted 24 h after acquisition training. Data are presented as the mean ± SEM (*n* = 7 rats per group). * *p* < 0.05 compared with the normal group; # *p* < 0.05 and ## *p* < 0.01 compared with the control (SCO-only) group. AR mixture, *Atractylodes macrocephala* Koidzumi and *Rumex acetosa* L. at a 2:1 ratio; Don, donepezil 2 mg/kg; PA, passive avoidance; SCO, scopolamine.

**Figure 5:**
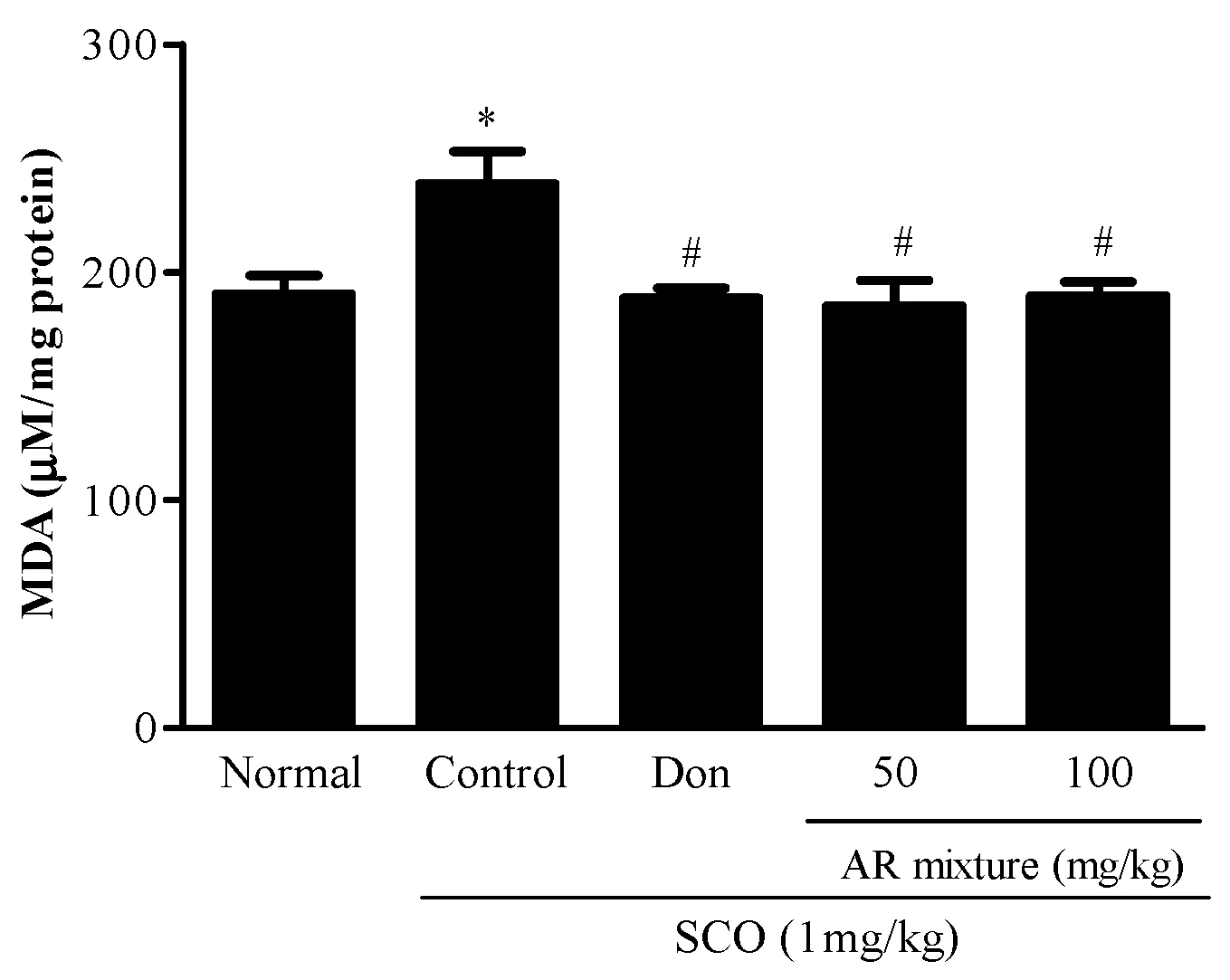
Effects of the AR mixture on hippocampal MDA levels in SCO-treated rats. Data are presented as the mean ± SEM (*n* = 7 rats per group). * *p* < 0.05 compared with the normal group; # *p* < 0.05 compared with the control (SCO-only) group. AR mixture, *Atractylodes macrocephala* Koidzumi and *Rumex acetosa* L. at a 2:1 ratio; Don, donepezil 2 mg/kg; MDA, malondialdehyde; SCO, scopolamine.

**Figure 6:**
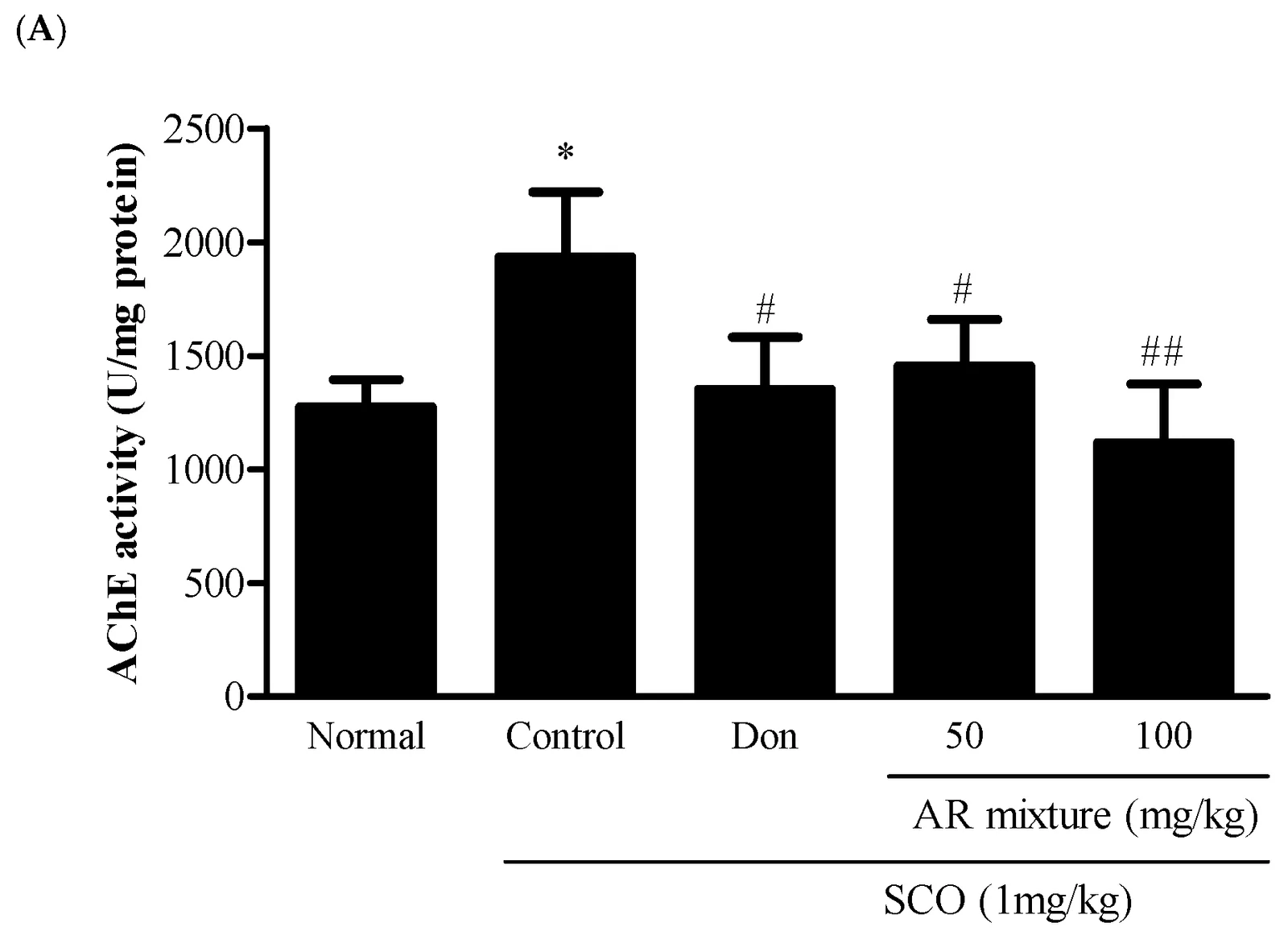

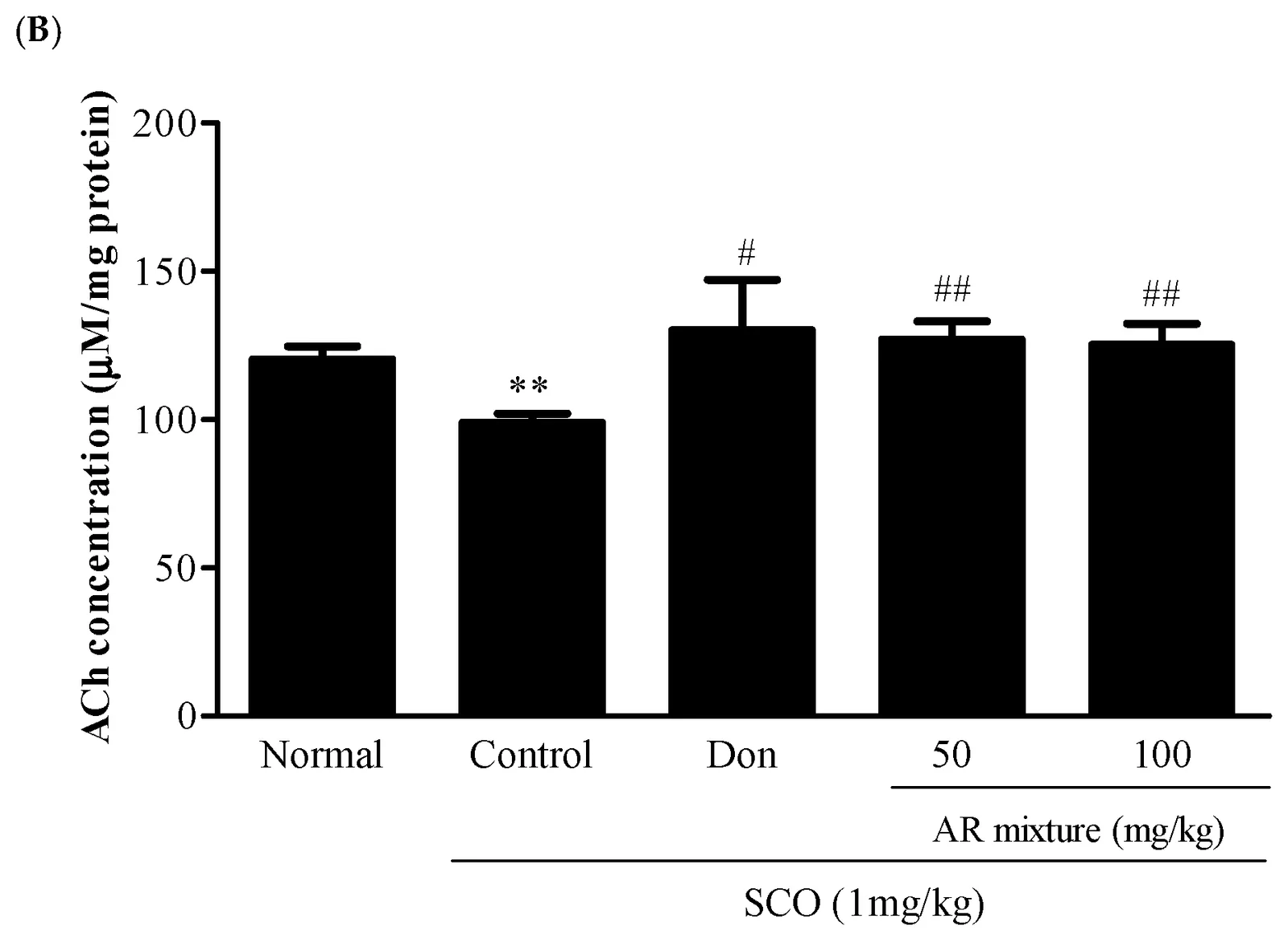
Effects of the AR mixture on hippocampal AChE activity (**A**) and ACh concentrations (**B**) in SCO-treated rats. Data are presented as the mean ± SEM (n = 7 rats per group). * *p* < 0.05 and ** *p* < 0.01 compared with the normal group; # *p* < 0.05 and ## *p* < 0.01 compared with the control (SCO-only) group. ACh, acetylcholine; AChE, acetylcholinesterase; AR mixture, *Atractylodes macrocephala* Koidzumi and *Rumex acetosa* L. at a 2:1 ratio; Don, donepezil 2 mg/kg; SCO, scopolamine.

**Figure 7:**
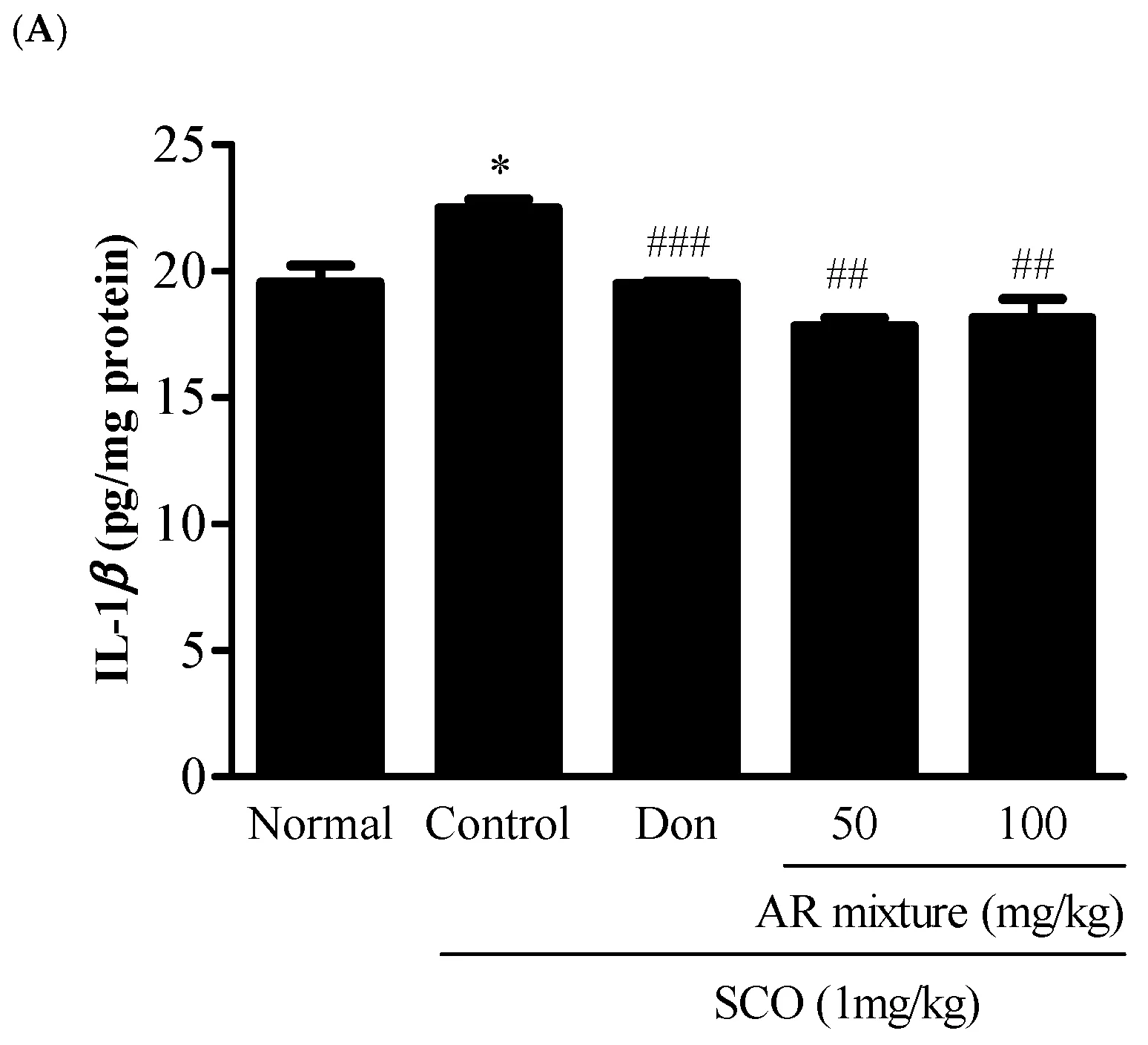

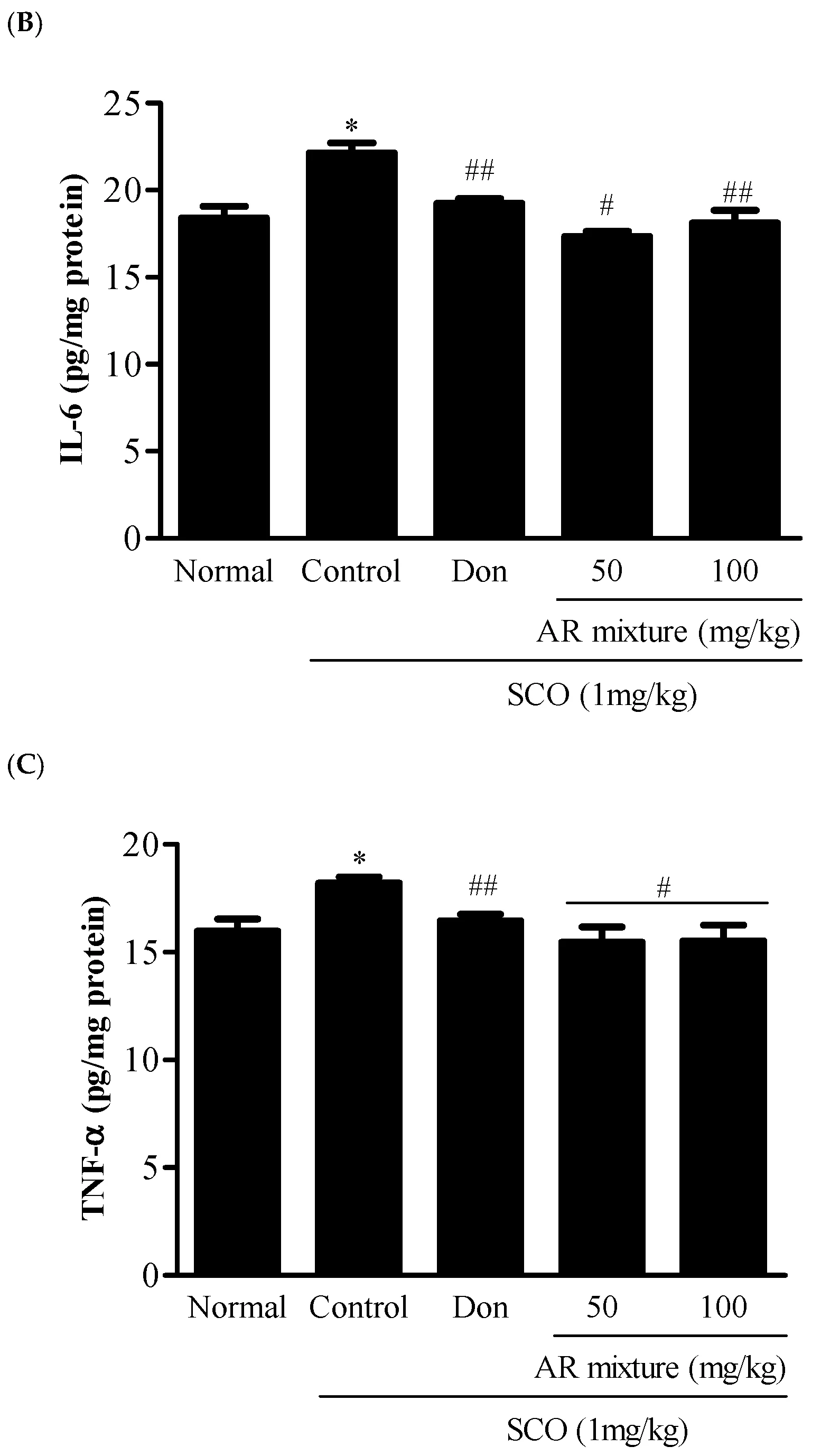
Effects of the AR mixture on hippocampal IL-1β (**A**), IL-6 (**B**), and TNF-α (**C**) concentrations in SCO-treated rats. Data are presented as the mean ± SEM (*n* = 7 rats per group). * *p* < 0.05 compared with the normal group; # *p* < 0.05, ## *p* < 0.01 and ### *p* < 0.001 compared with the control (SCO-only) group. AR mixture, *Atractylodes macrocephala* Koidzumi. and *Rumex acetosa* L. at a 2:1 ratio; Don, donepezil 2 mg/kg; IL-1β, interleukin-1beta; IL-6, interleukin-6; SCO, scopolamine; TNF-α, tumor necrosis factor-alpha.

**Figure 8:**
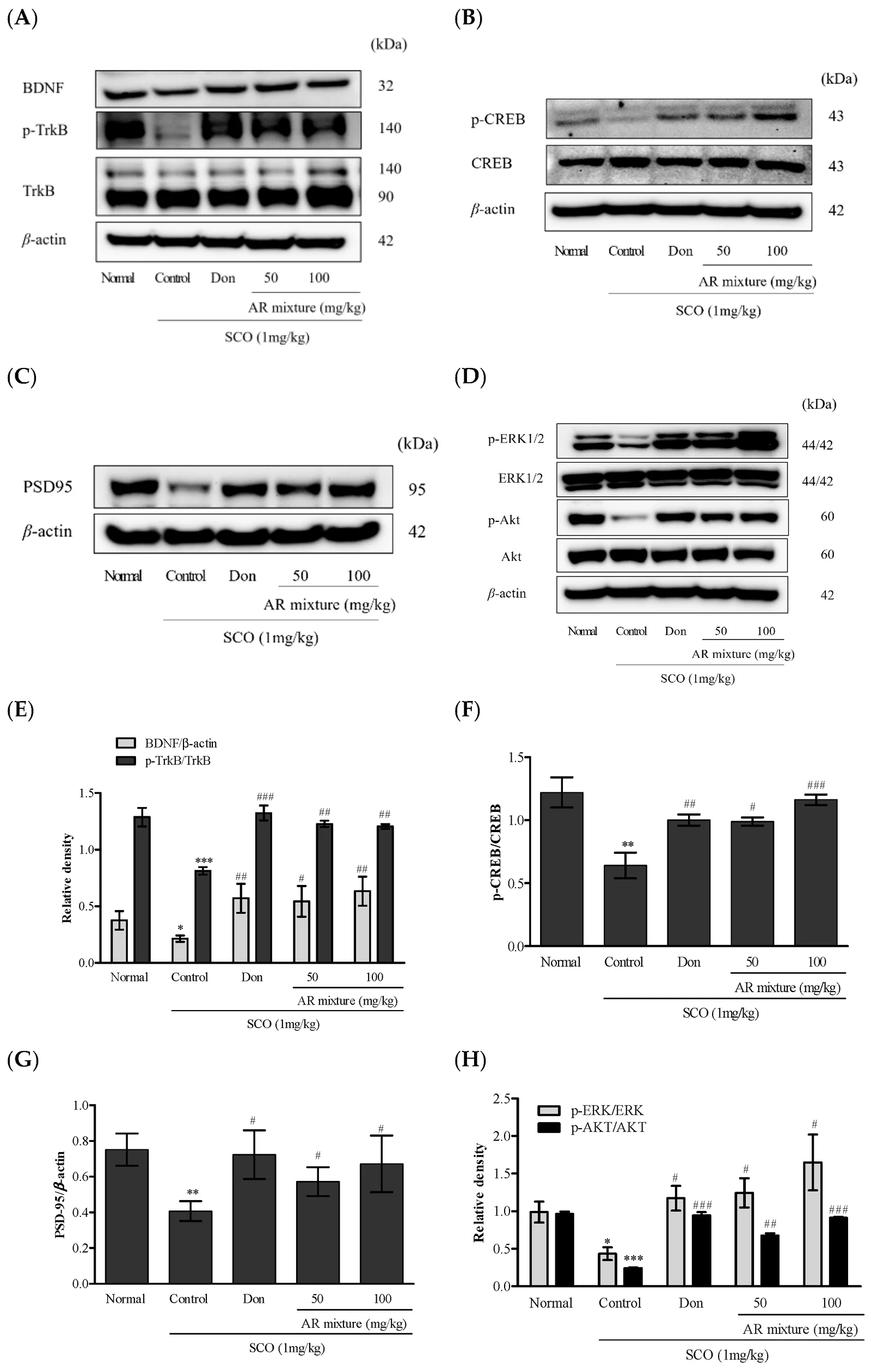
Effects of the AR mixture on hippocampal BDNF/TrkB/CREB signaling, PSD-95 expression, and ERK1/2 and Akt phosphorylation in SCO-treated rats. (**A**–**D**) Representative Western blots of hippocampal (**A**) BDNF, p-TrkB, and t-TrkB; (**B**) p-CREB and t-CREB; (**C**) PSD-95; and (**D**) p-ERK1/2, t-ERK1/2, p-Akt, and t-Akt. β-Actin was used as the loading control. (**E**–**H**) Densitometric analyses of the corresponding protein-expression levels and phosphorylation ratios. Data are presented as the mean ± SEM (*n* = 7 rats per group). * *p* < 0.05, ** *p* < 0.01 and *** *p* < 0.001 compared with the normal group; # *p* < 0.05, ## *p* < 0.01 and ### *p* < 0.001 compared with the control (SCO-only) group. AR mixture, *Atractylodes macrocephala* Koidzumi. and *Rumex acetosa* L. at a 2:1 ratio; BDNF, brain-derived neurotrophic factor; CREB, cAMP-response element binding protein; Don, donepezil 2 mg/kg; ERK1/2, extracellular signal-regulated kinase 1/2; Akt, protein kinase B; PSD-95, postsynaptic density protein-95; SCO, scopolamine; TrkB, tropomyosin receptor kinase B.

**Figure 9:**
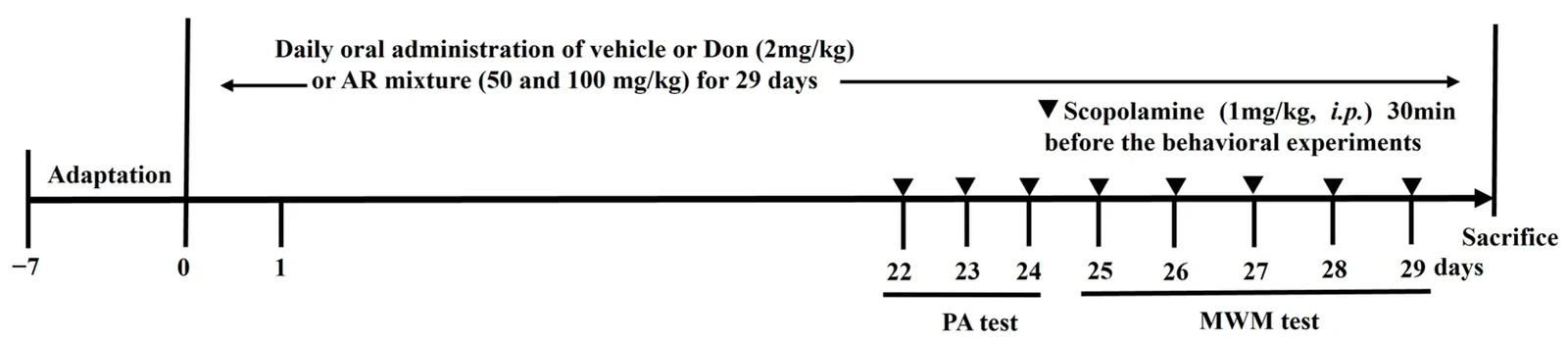
Experimental timeline of drug administration and behavioral assessments.

## Data Availability

The data presented in this study are available on request from the corresponding author due to restrictions related to technology transfer and commercialization.

## Supplementary Materials

The following supporting information can be downloaded at https://www.mdpi.com/article/10.3390/ijms27219410/s1.

## Abbreviations

ACh	acetylcholine
AChE	acetylcholinesterase
AD	Alzheimer’s disease
Akt	protein kinase B
AM	*Atractylodes macrocephala* Koidzumi
AR mixture	2:1 mixture of *Atractylodes macrocephala* and *Rumex acetosa* extracts
BDNF	brain-derived neurotrophic factor
CREB	cAMP response element-binding protein
ELISA	enzyme-linked immunosorbent assay
ERK1/2	extracellular signal-regulated kinase 1/2
HPLC	high-performance liquid chromatography
HRP	horseradish peroxidase
IL-1β	interleukin-1 beta
IL-6	interleukin-6
MDA	malondialdehyde
MPLC	medium-pressure liquid chromatography
MWM	Morris water maze
NMR	nuclear magnetic resonance
NR2	*N*-methyl-D-aspartate receptor 2
PA	passive avoidance
PSD-95	postsynaptic density protein-95
RA	*Rumex acetosa* L.
SCO	scopolamine
TNF-α	tumor necrosis factor-alpha
TrkB	tropomyosin receptor kinase B
