# Supplementary material for: Cognitive-Enhancing and Neuroprotective Effects of a Combination Extract of Atractylodes macrocephala and Rumex acetosa Against Scopolamine-Induced Memory Impairment
Source: Int J Mol Sci. 2026 Oct 22;27(21):9410. doi: 10.3390/ijms27219410 (PMC13762987; doi:10.3390/ijms27219410)
Supplement: Supplementary file 1 [file ijms-27-09410-s001.zip › final_Supplementary_Data.pdf]

Cognitive-Enhancing and Neuroprotective Effects of a Combination Extract of *Atractylodes macrocephala* and *Rumex acetosa* against Scopolamine-Induced Memory Impairment

Dool-Ri Oh[1], Hwan Lee[1], Yonguk Kim[1], Yujin Kim[1] and Donghyuck Bae[1,*]

Supplementary Table S1. HPLC-PDA gradient elution conditions.

Time (min)	Solvent A (acetonitrile, %)	Solvent B (water, %)
0–7	7	93
7–10	7–15	85–93
10–20	15–20	80–85
20–21	20–55	45–80
21–32	55–65	35–45
32–34	65	35

Supplementary Table S2. Statistical assumption checks, omnibus ANOVA, and Tukey-adjusted p-values for selected pairwise comparisons.

Figure / Endpoint	n	Shapiro–Wilk (p)	Brown–Forsythe (p)	Omnibus ANOVA F(df1, df2)	ANOVA p	Tukey-Adjusted p (Control vs SCO)	Tukey-Adjusted p (SCO vs AR 2:1 30μg/mL)	Tukey-Adjusted p (SCO vs AR 2:1 50μg/mL)
**2A** SCO conc.–cell viability	3	0.4125	0.6582	F(5, 12) = 38.41	<0.0001	<0.0001	—	—
**2C** Cell viability (50 μg/mL)	3	0.2841	0.5144	F(6, 14) = 14.85	0.0001	0.0003	0.0112	0.0008
**2E** p-CREB/CREB (30μg/mL)	3	0.6247	0.4891	F(6, 14) = 8.12	0.0006	0.0015	0.0019	—

Figure / Endpoint	n	Shapiro–Wilk (p)	Brown–Forsythe (p)	Omnibus ANOVA F(df1, df2)	ANOVA p	Tukey-Adjusted p (Normal vs SCO)	Tukey-Adjusted p (SCO vs AR 50mg/kg)	Tukey-Adjusted p (SCO vs AR 100mg/kg)
**3A** MWM: First trials (Day 1)	7	0.2941	0.4102	F(4, 30) = 8.54	0.0001	0.0032	0.0415	0.1250
**3A** MWM: First trials (Day 2)	7	0.3415	0.5124	F(4, 30) = 14.12	<0.0001	0.0006	0.0241	0.0005

**3A** MWM: first trials(Day3)	7	0.3142	0.4852	F(4, 30) = 16.84	<0.0001	0.0005	0.0315	0.0008
**3B** MWM: last trials(Day1)	7	0.4125	0.3841	F(4, 30) = 11.24	<0.0001	0.0008	0.0072	0.0004
**3B** MWM: last trials(Day2)	7	0.3841	0.6102	F(4, 30) = 13.95	<0.0001	0.0003	0.0415	0.0032
**3B** MWW: last trials(Day3)	7	0.4512	0.5214	F(4, 30) = 19.42	<0.0001	0.0002	0.0089	0.0005
**3D** Target-quadrant distance	7	0.3541	0.7412	F(4, 30) = 22.45	<0.0001	<0.0001	0.0028	0.0031
**3E** Target-quadrant time	7	0.5812	0.6120	F(4, 30) = 5.84	0.0014	0.0042	0.0381	0.0006
**4** Passive avoidance latency	7	0.1985	0.3241	F(4, 30) = 18.92	<0.0001	<0.0001	0.0011	0.0034
**5** MDA	7	0.7415	0.8124	F(4, 30) = 4.15	0.0085	0.0125	0.0241	0.0195
**6A** AChE activity	7	0.4952	0.5641	F(4, 30) = 6.21	0.0010	0.0048	0.0315	0.0015
**6B** ACh concentration	7	0.6102	0.4358	F(4, 30) = 5.12	0.0029	0.0089	0.0042	0.0031

**7A** IL-1β	7	0.3341	0.5217	F(4, 30) = 7.84	0.0002	0.0011	0.0031	0.0031
**7B** IL-6	7	0.5147	0.6841	F(4, 30) = 6.95	0.0005	0.0024	0.0221	0.0042
**7C** TNF-α	7	0.4219	0.7102	F(4, 30) = 4.38	0.0068	0.0142	0.0401	0.0188
**8E** BDNF/β-actin	7	0.6852	0.5941	F(4, 30) = 3.98	0.0104	0.0215	0.0392	0.0094
**8E** p-TrkB/TrkB	7	0.2914	0.4125	F(4, 30) = 9.42	<0.0001	0.0008	0.0035	0.0011
**8F** p-CREB/CREB (In vivo)	7	0.5412	0.6321	F(4, 30) = 6.18	0.0011	0.0039	0.0142	0.0021
**8G** PSD-95	7	0.4105	0.4852	F(4, 30) = 4.85	0.0039	0.0068	0.0410	0.0115
**8H** p-ERK/ERK	7	0.7124	0.5241	F(4, 30) = 3.54	0.0176	0.0298	0.0482	0.0199
**8H** p-Akt/Akt	7	0.3654	0.6124	F(4, 30) = 11.24	<0.0001	0.0005	0.0024	0.0009

Note: Entries are reported as Tukey-adjusted p-values based on the analysis of variance results. Normality of residuals was verified using the Shapiro–Wilk test, and homogeneity of variances was verified using the Brown–Forsythe test. Figure 2 includes in vitro comparisons and does not use the same treatment groups as the in vivo panels.

Contents

Supplementary Figure S1. ^{1}H-NMR spectrum (CD_3OD) of **compound 1**

Supplementary Figure S2. ^{13}C-NMR spectrum (CD_3OD) of **compound 1**

Supplementary Figure S3. COSY-NMR spectrum (CD_3OD) of **compound 1**

Supplementary Figure S4. HSQC spectrum (CD_3OD) of **compound 1**

Supplementary Figure S5. HMBC spectrum (CD_3OD) of **compound 1**

Supplementary Figure S6. ^{1}H-NMR spectrum (CD_3OD) of **compound 2**

Supplementary Figure S7. ^{13}C-NMR spectrum (CD_3OD) of **compound 2**

Supplementary Figure S8. DEPT spectrum (CD_3OD) of **compound 2**

Supplementary Figure S9. COSY-NMR spectrum (CD_3OD) of **compound 2**

Supplementary Figure S10. HMQC spectrum (CD_3OD) of **compound 2**

Supplementary Figure S11. HMBC spectrum (CD_3OD) of **compound 2**

Supplementary Figure S12. ^{1}H-NMR spectrum (CD_3OD) of **compound 3**

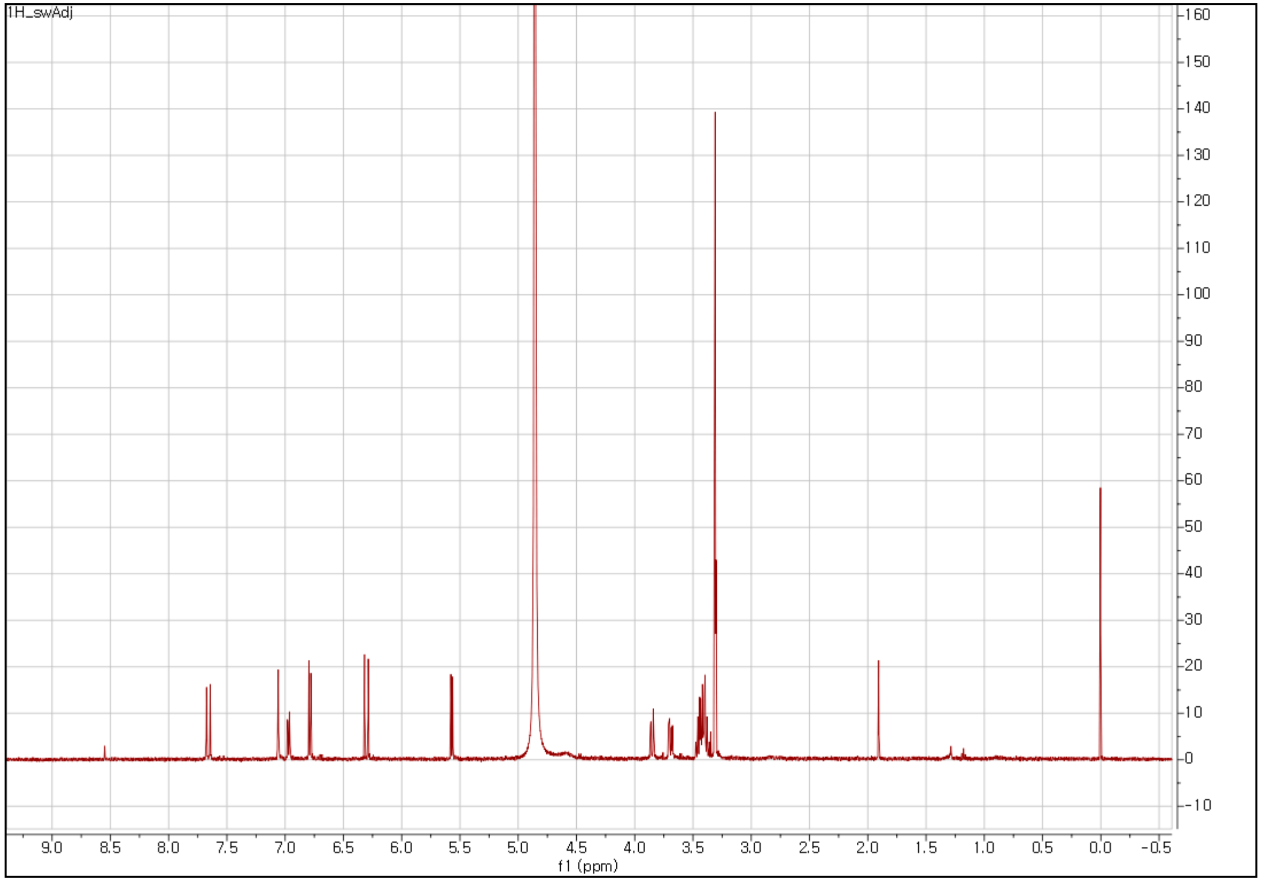

Supplementary Figure S1. ^{1}H-NMR spectrum (CD_3OD) of **compound 1**

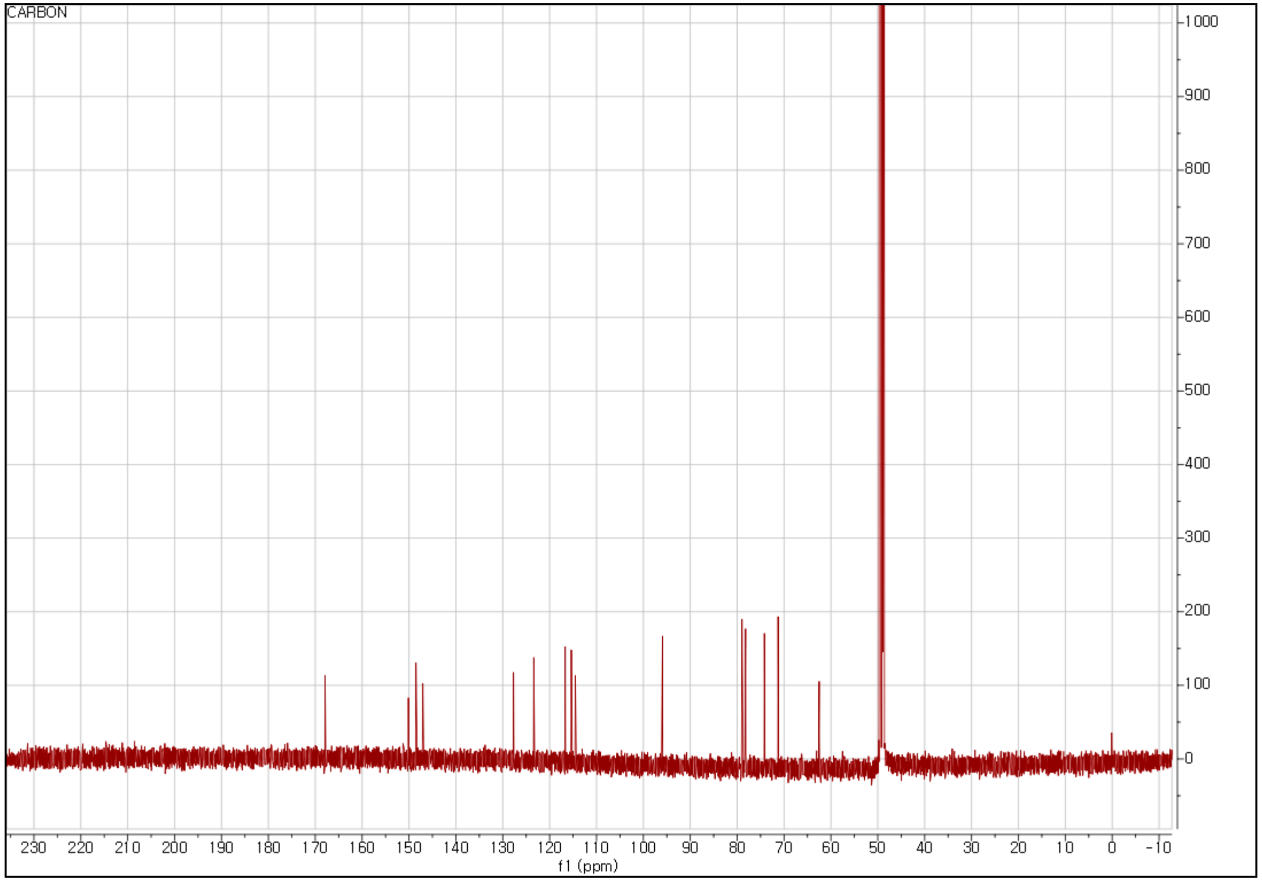

Supplementary Figure S2. ^{13}C-NMR spectrum (CD_3OD) of **compound 1**

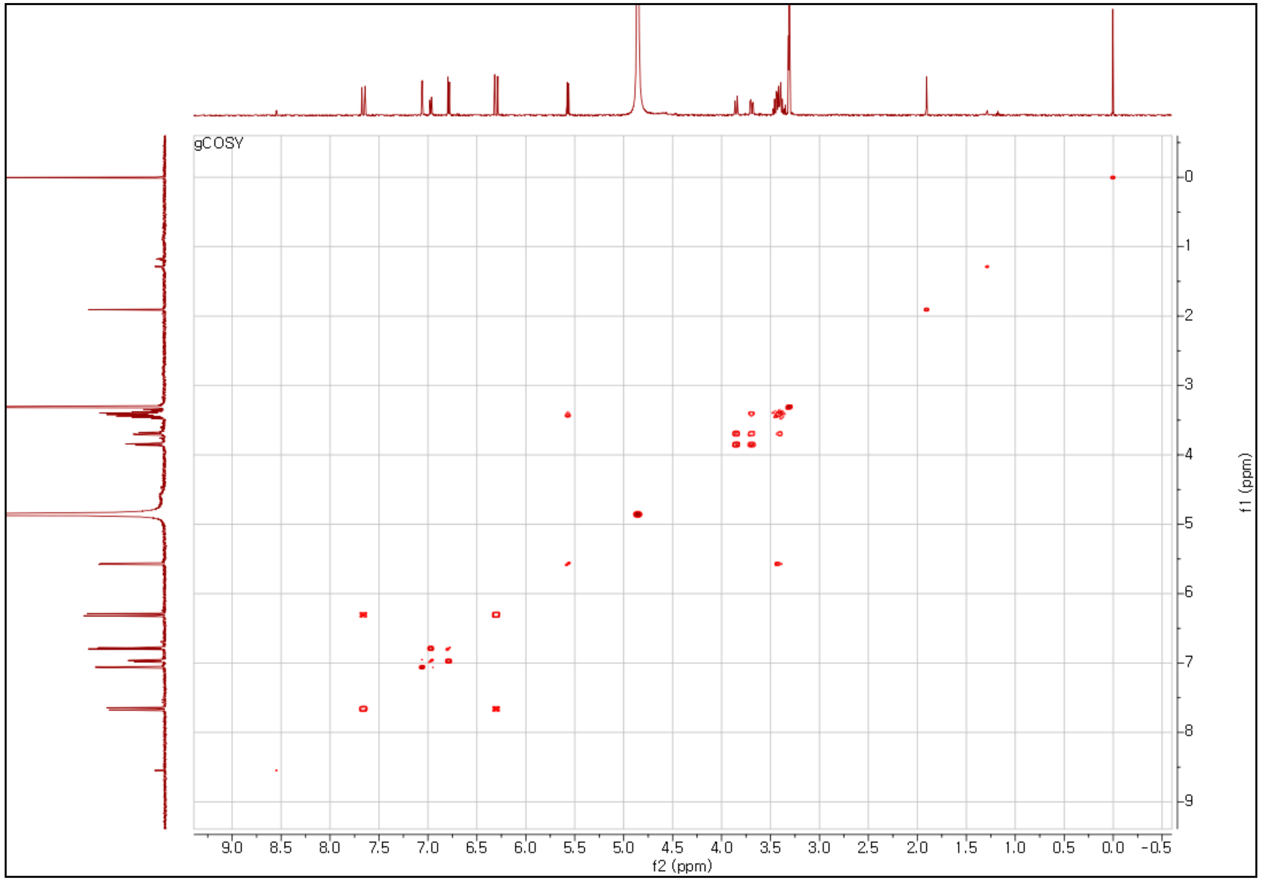

Supplementary Figure S3. COSY-NMR spectrum (CD_3OD) of **compound 1**

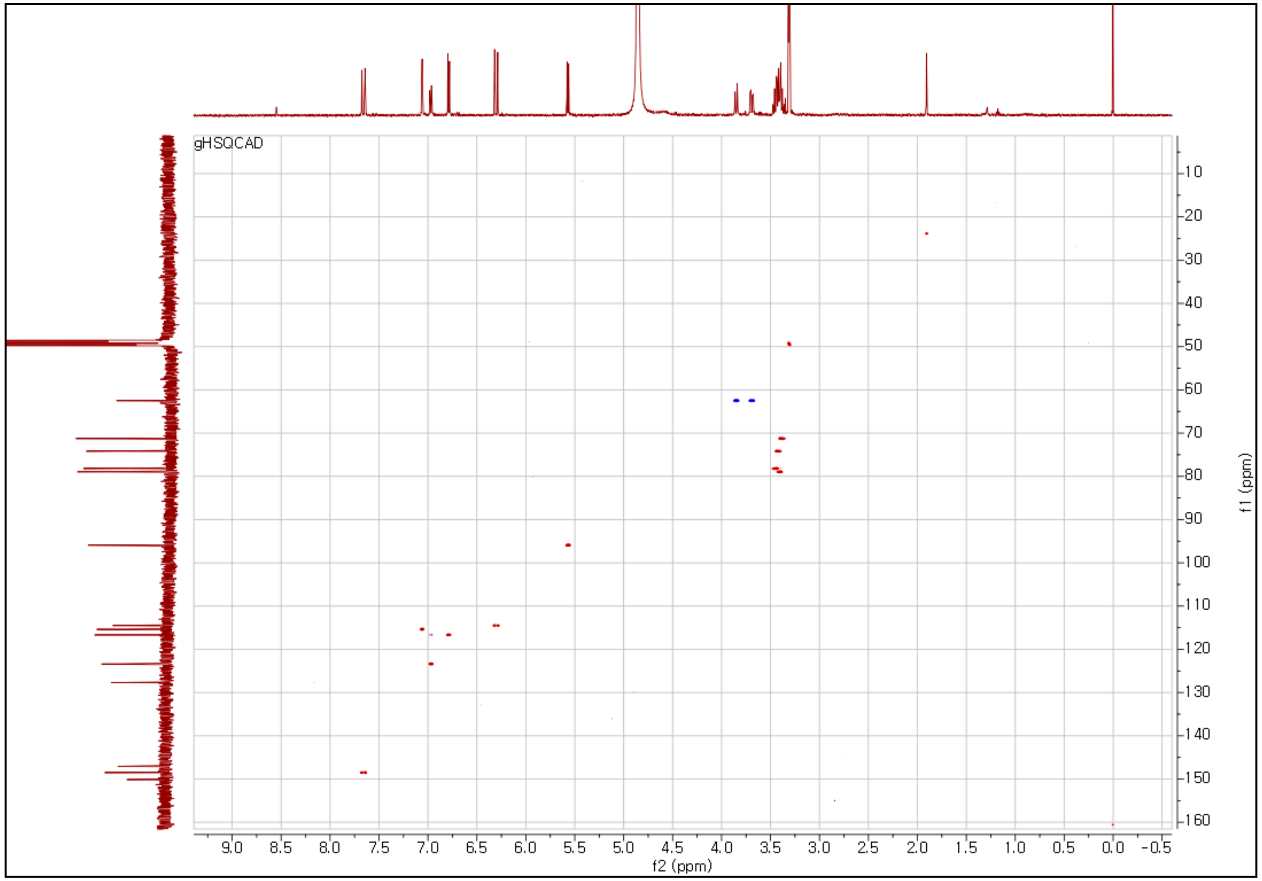

Supplementary Figure S4. HSQC spectrum (CD_3OD) of **compound 1**

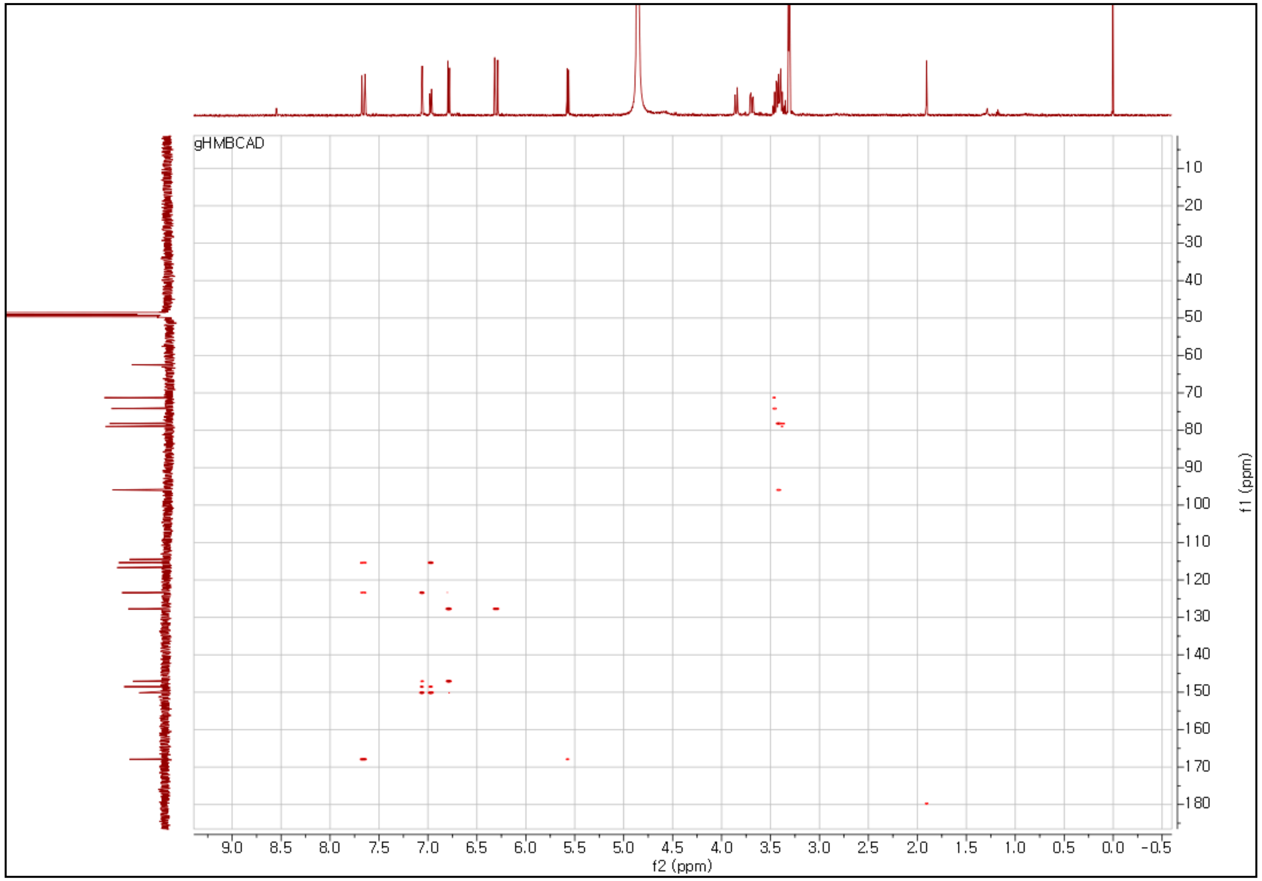

Supplementary Figure S5. HMBC spectrum (CD_3OD) of **compound 1**

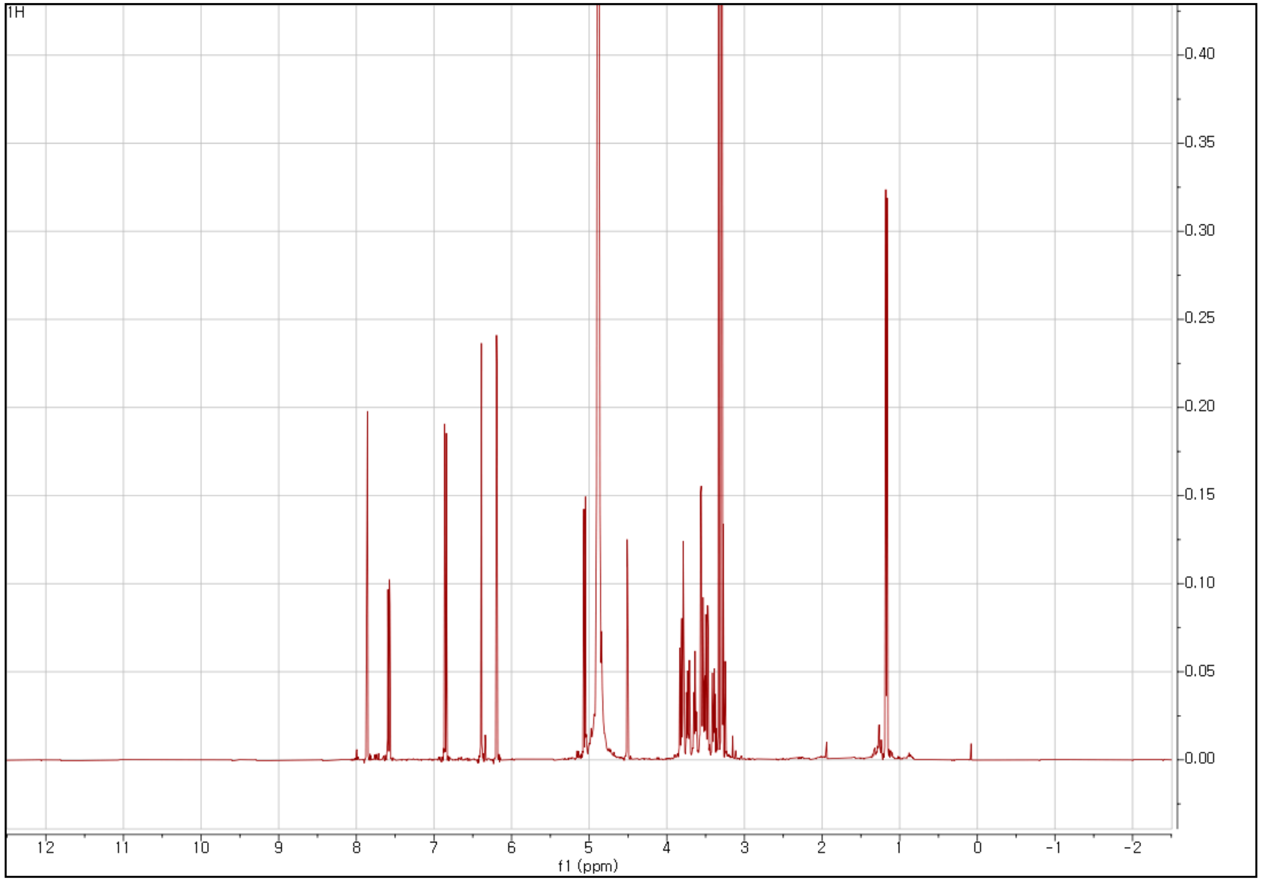

Supplementary Figure S6. ^{1}H-NMR spectrum (CD_3OD) of **compound 2**

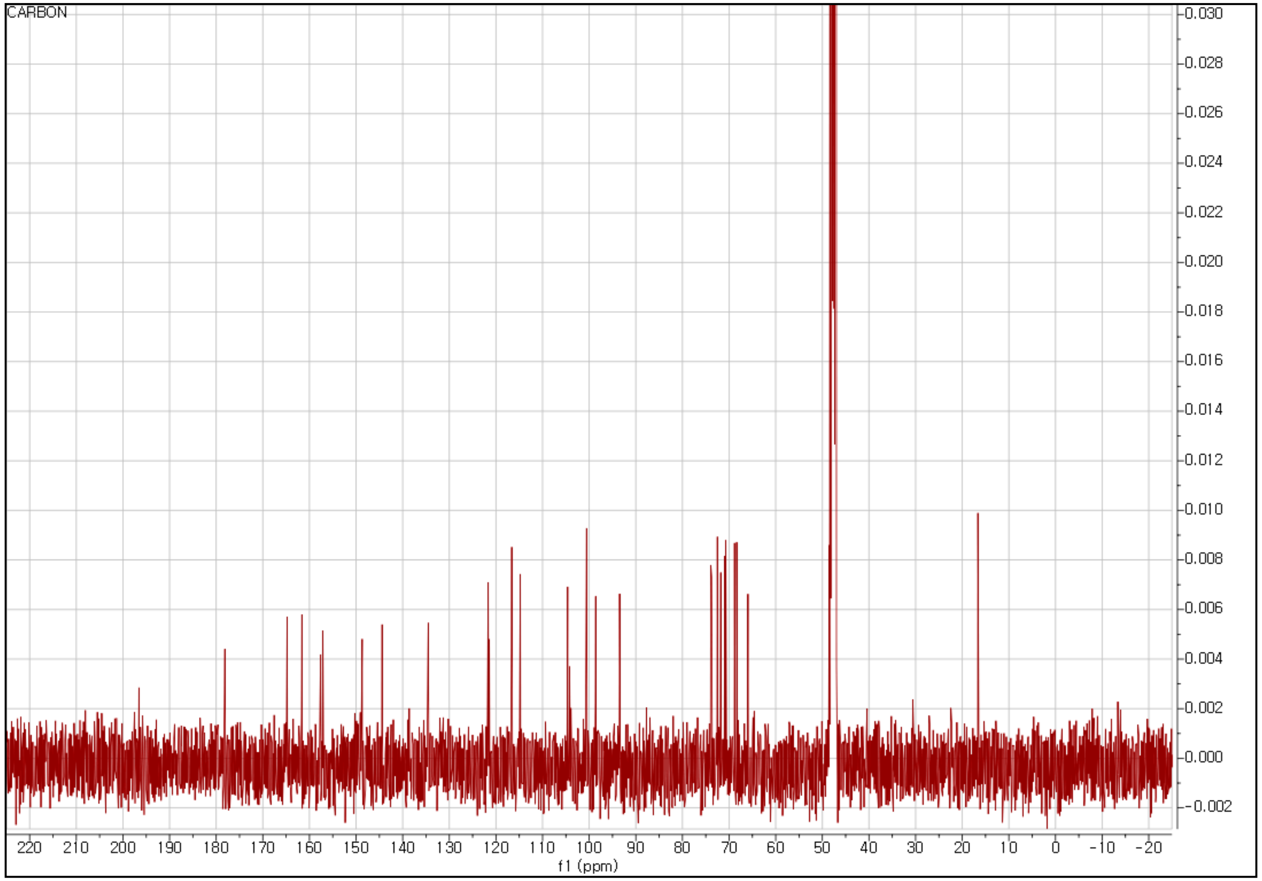

Supplementary Figure S7. ^{13}C-NMR spectrum (CD_3OD) of **compound 2**

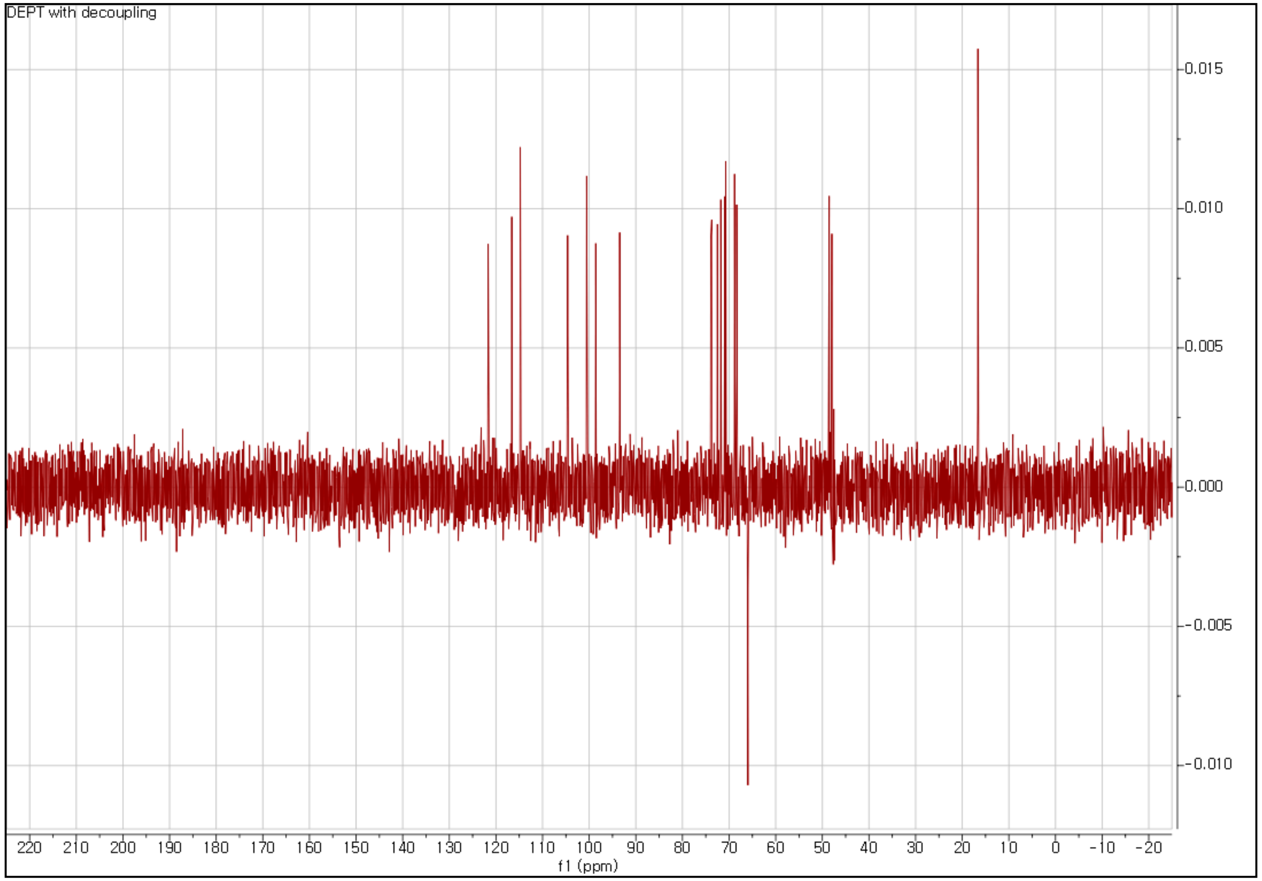

Supplementary Figure S8. DEPT spectrum (CD_3OD) of **compound 2**

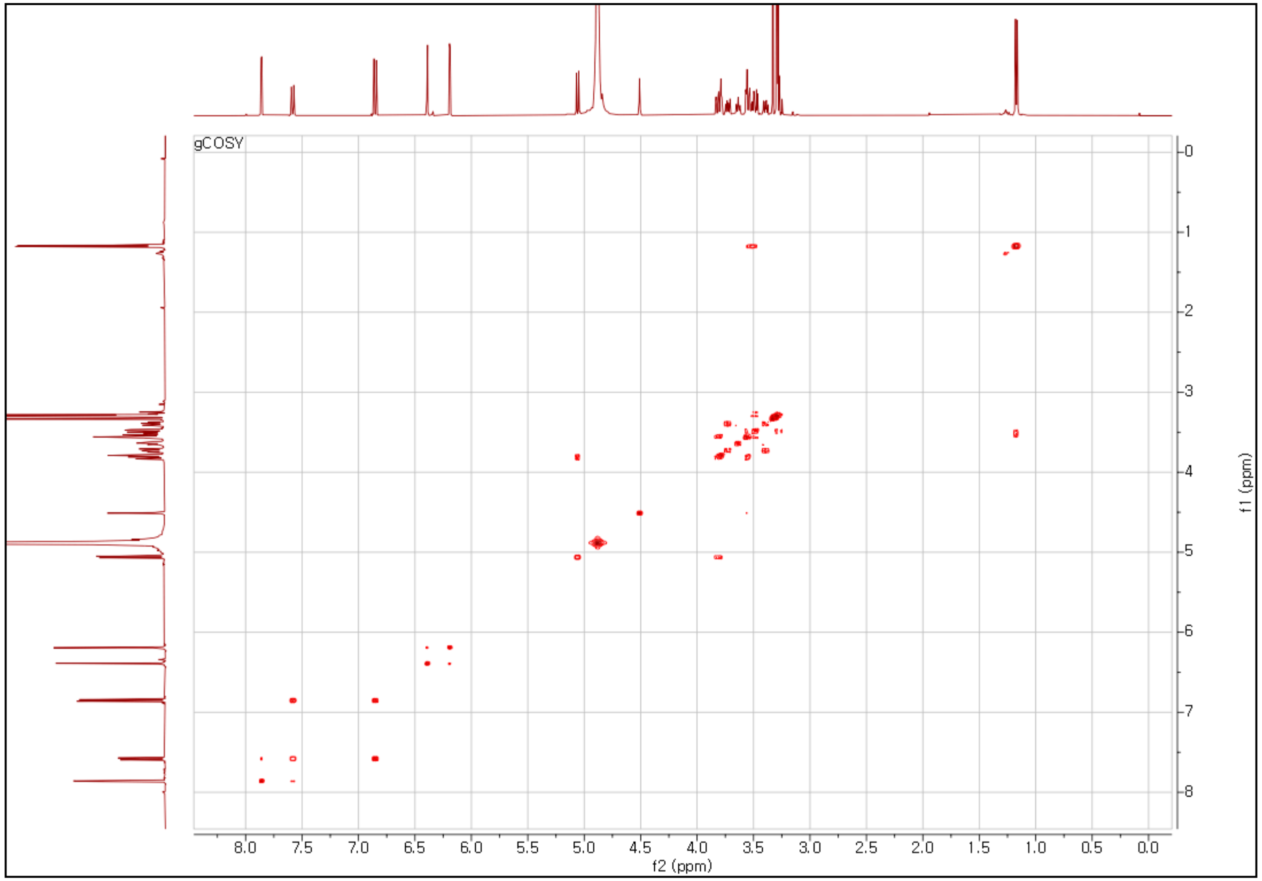

Supplementary Figure S9. COSY-NMR spectrum (CD_3OD) of **compound 2**

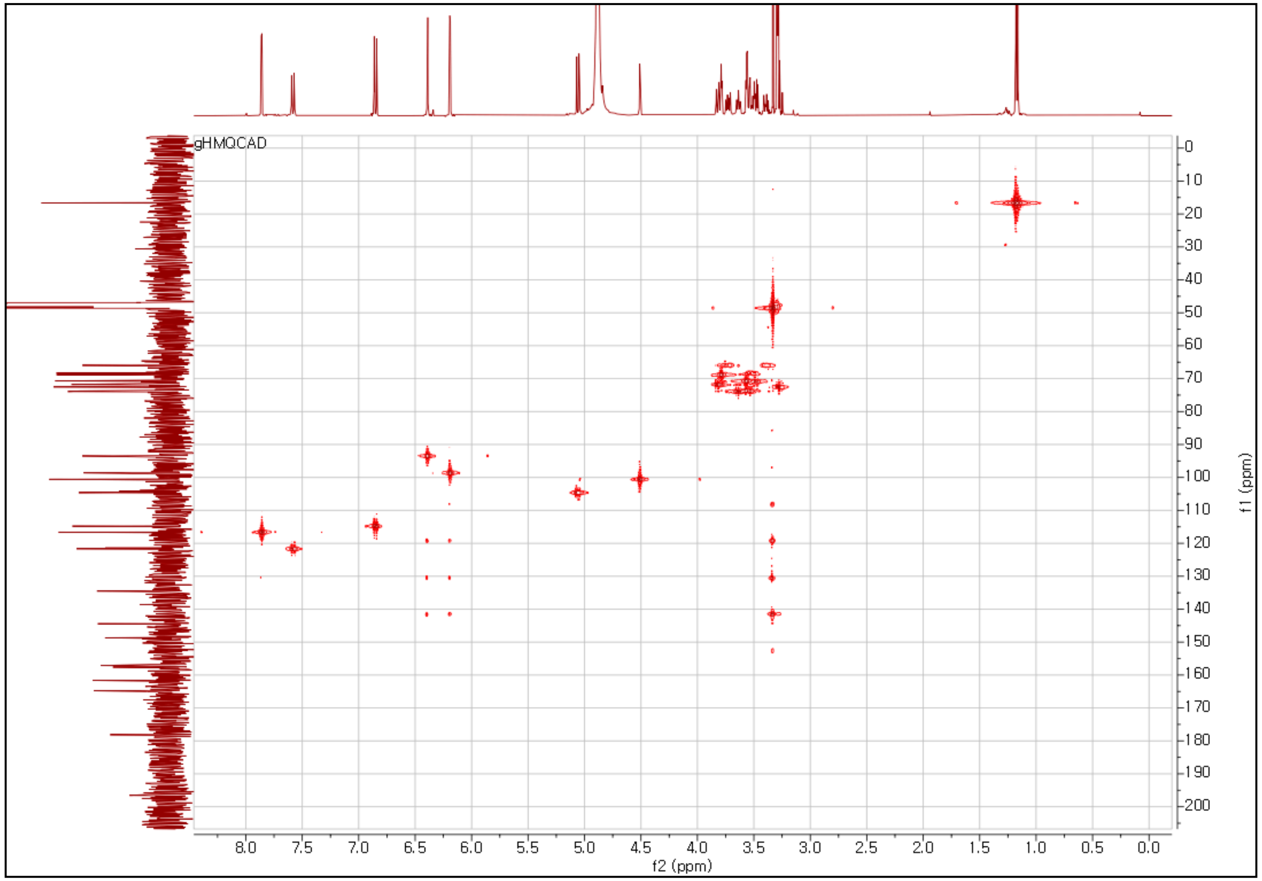

Supplementary Figure S10. HMQC spectrum (CD_3OD) of **compound 2**

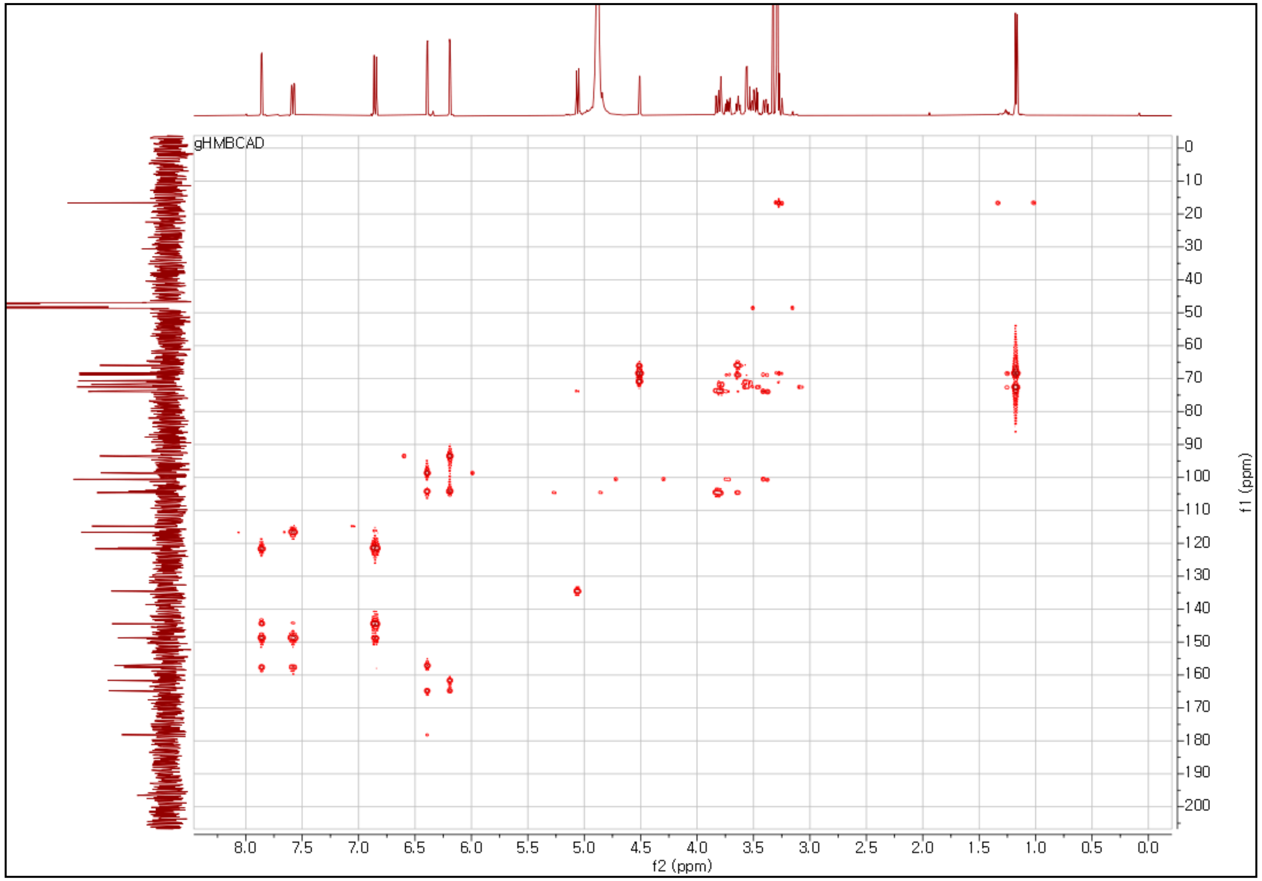

Supplementary Figure S11. HMBC spectrum (CD_3OD) of **compound 2**

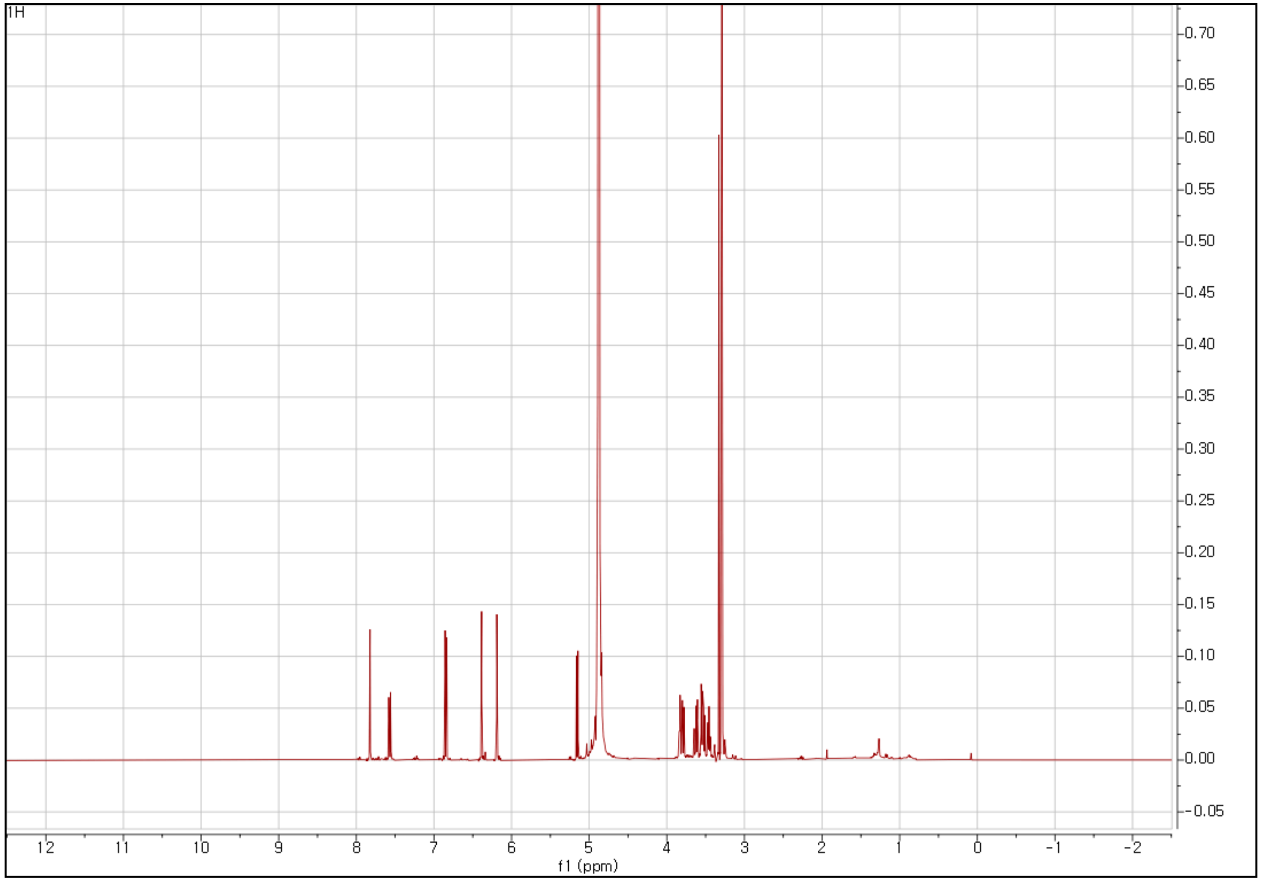

Supplementary Figure S12. ^{1}H-NMR spectrum (CD_3OD) of **compound 3**
